# Comparative study of the direct and photocatalytic light activation of platinum(iv) prodrugs†

**DOI:** 10.1039/d6dt01745b

**Published:** 2026-10-12

**Authors:** Kinjal Mileche, Susana M. Meza Huaman, Ke Wu, Jake A. Vickery, Hannah Coxhead, Alexander N. Smith-Xu, Patricia Juhl Allworthy, Jovana Katrinka-Mavrak, Aminesena Baser, Safacan Kolemen, Manuel M. Müller, Mahvash Tavassoli, Ismael Diez-Perez, Eleanor Stride, John F. Callan, Adam C. Sedgwick

**Affiliations:** a Department of Chemistry, King's College London 7 Trinity Street London SE1 1DB, UK adam.sedgwick@kcl.ac.uk; b Centre for Host–Microbiome Interactions, King's College London SE1 1UL, London UK; c Institute of Biomedical Engineering, Department of Engineering Sciences, Old Road Campus Research Building, University of Oxford Headington Oxford UK; d Department of Chemistry, Koç University Istanbul 34450 Türkiye; e Biomedical Sciences Research Institute, University of Ulster Coleraine Northern Ireland UK j.callan@ulster.ac.uk; f Graduate School of Health Sciences, Cellular and Molecular Medicine Program, Koç University Istanbul, 34450 Turkey

## Abstract

Here, we present a comparative study of direct and photocatalyst-mediated photoactivation of reporter-functionalised Pt(iv) prodrugs, Carbo-4NP and CarboNap. Direct blue-light irradiation and green-light activation using the photocatalyst Rose Bengal both achieved rapid and complete Pt(iv) reduction in solution, monitored by UV-Vis and fluorescence spectroscopy. Translation to live cells revealed a marked divergence between the two approaches. Direct blue light (415 nm) photoactivation produced >300-fold enhancement in fluorescence intensities upon probe activation and >10-fold in cytotoxicity. Photocatalytic activation was considerably less effective in cells despite its excellent solution-phase performance. These findings identify direct photoactivation as the superior strategy for intracellular activation of Pt(iv) prodrugs and support the further development of direct light-activated platinum therapeutics.

## Abstract 2

Here, we present a comparative study of direct and photocatalyst-mediated photoactivation of reporter-functionalised Pt(iv) prodrugs, Carbo-4NP and CarboNap.

## Graphical abstract

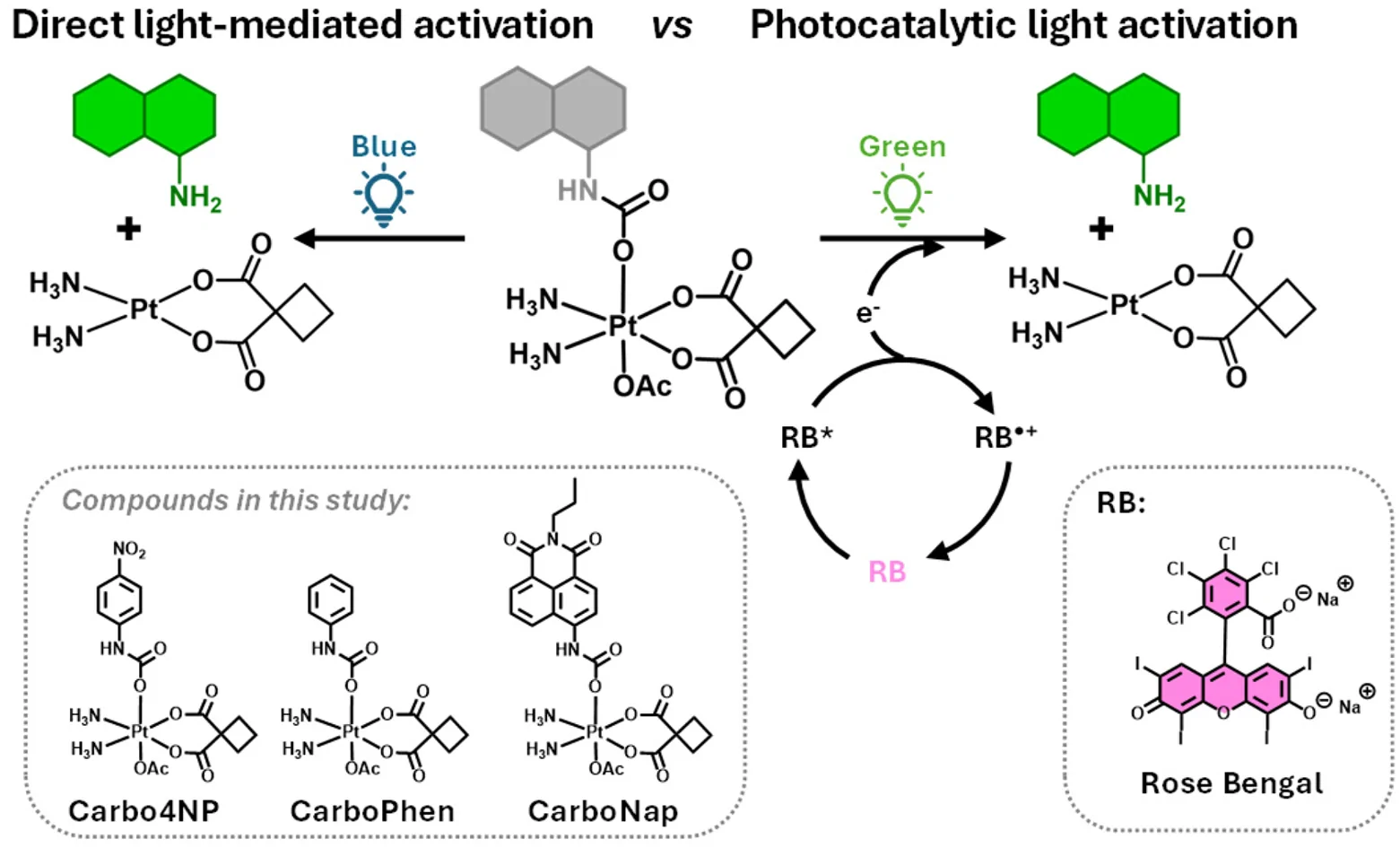

Platinum(iv)-based prodrugs have emerged as promising alternatives to conventional Pt(ii)-based chemotherapeutics.^1–4^ In recent years, light-mediated activation of Pt(iv) prodrugs has attracted increasing attention as a strategy for achieving precise spatiotemporal control over the release of cytotoxic Pt(ii) species.^5,6^ To date, two light-based approaches have been explored: (1) direct photoactivation of Pt(iv) prodrugs incorporating chromophores or photoresponsive functional groups,^6–9^ and (2) indirect activation of Pt(iv) prodrugs through the photoexcitation of an external photocatalyst.^10–13^ Although both strategies have shown considerable promise, they have not been systematically compared. Here, we utilise our colorimetric and fluorometric Pt(iv) reporter methodologies^14–16^ to evaluate these two approaches. Direct photoactivation was investigated using the reporter-functionalised Pt(iv) complexes, Carbo-4NP and CarboNap,^6^ while, Rose Bengal (RB) was selected as the model catalyst for indirect activation of the corresponding complexes, owing to its well-established photochemical properties and due to our ongoing interest in RB and its derivatives for clinical applications (Scheme 1).^17–19^

**Scheme 1:**
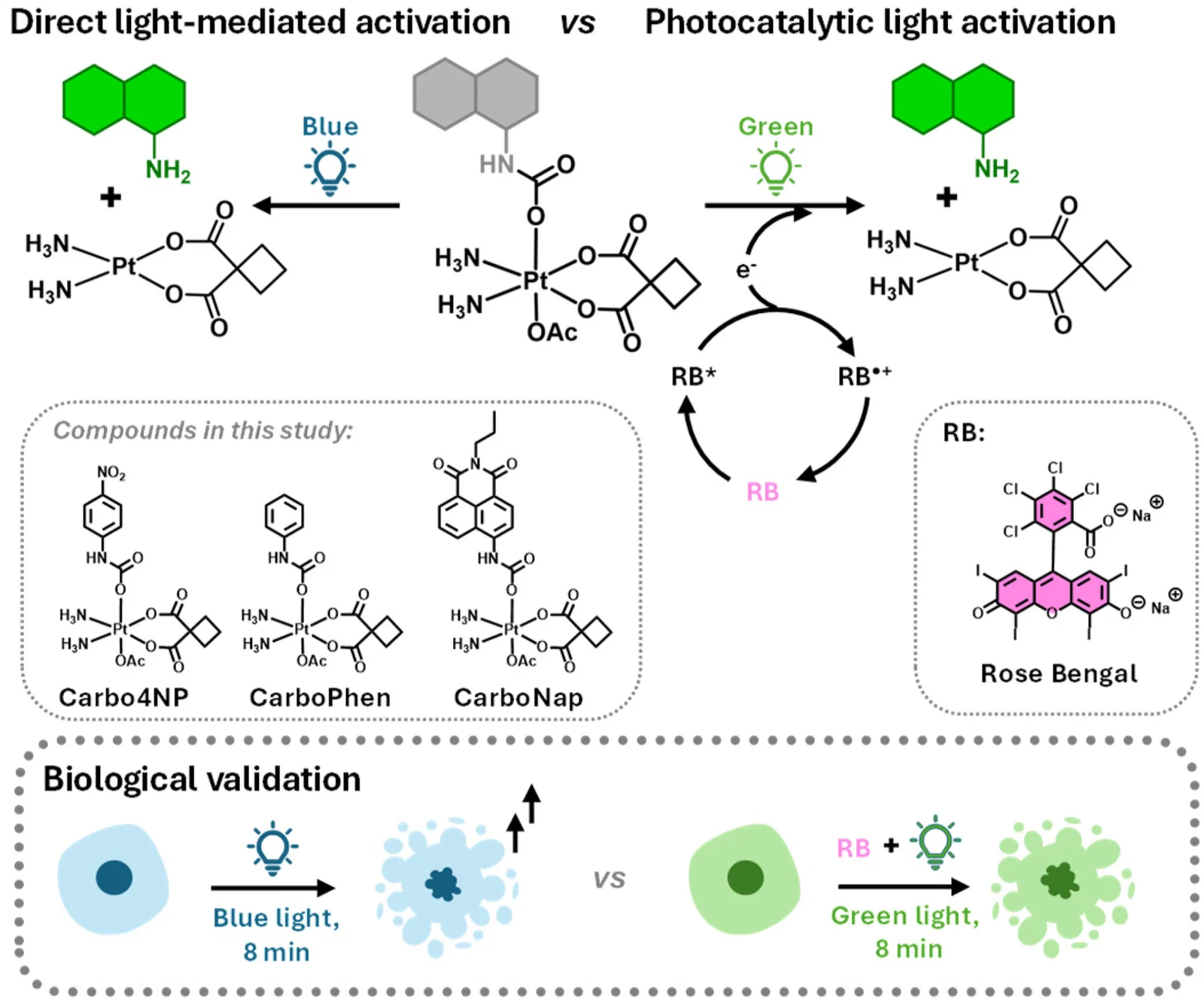
General schematic of the proposed study, comparing direct *vs.* photocatalytic light-mediated activation of Pt(iv) prodrugs with photocatalyst Rose Bengal (RB), and including the chemical structure of Pt(iv) complexes, CarboPhen, Carbo-4NP and CarboNap investigated in this manuscript.

RB possesses well-established photosensitising and photocatalytic properties.^20^ Given the approximate literature values for excited-state oxidation and reduction potentials (+1.18 and −1.33 V *vs.* SCE, respectively),^21^ we hypothesised that either oxidative or reductive quenching pathways could promote the photocatalytic reduction of Pt(iv) prodrugs upon light irradiation. Supporting this hypothesis, a previous mechanistic study of potassium hexachloroplatinate(iv) (K_2_[PtCl_6_]) and RB reported the photobleaching of RB only in the presence of Pt(iv), consistent with a photoinduced electron-transfer process.^22^ K_2_[PtCl_6_] serves as a useful synthetic precursor to clinically relevant Pt(iv) prodrugs but it is not itself a therapeutically relevant Pt(iv) complex. These findings, therefore, warrant a more comprehensive investigation of the excited-state interactions between RB and clinically relevant Pt(iv) prodrugs.

## Results and discussion

Our previous studies, together with literature reports, have demonstrated that carboplatin-derived Pt(iv) prodrugs display excellent stability towards reduction and aqueous hydrolysis.^2,6,23^ These complexes therefore provide a stringent platform for comparing direct and indirect activation strategies. Our previously reported colorimetric 4-nitrophenyl (4-NP)-functionalised carboplatin-based Pt(iv) complex (Carbo-4NP) was selected as the initial model system. Under UV light irradiation, Pt(iv) reduction generates 4-nitroaniline, enabling the reaction to be monitored by HPLC and UV-Vis spectroscopy through a characteristic absorption shift from 330 to 380 nm.^15^

Control studies were first performed. Experiments were initially performed in deionised water (DI) to minimise potential interference from phosphate-buffered saline (PBS) and HEPES buffer. Incubation of Carbo-4NP (50 µM) with sodium ascorbate (NaAsc, 1 mM) alone resulted in no change to its UV-Vis absorption spectrum (Fig. S1). Similarly, irradation of Carbo-4NP (50 µM), with green light irradiation (520 nm, 25.1 mW cm^−2^) in the absence of RB produced no detectable spectral changes (Fig. S2). Next, a solution containing Carbo-4NP (50 µM) and RB (5 µM) was irradiated under identical conditions, and UV-Vis spectra were recorded at 15 s intervals. Consistent with the previous report,^22^ the characteristic absorption band of RB at 550 nm decreased markedly over 120 s, while minimal changes were observed to the absorption bands associated with Carbo-4NP (Fig. 1a). The reaction mixture was also found to change from pink to colourless, consistent with RB photobleaching^22^ (Fig. S3). Irradiation of RB alone under identical conditions (2 min, 520 nm, 25.1 mW cm^−2^) produced no change in its characteristic absorption band (Fig. S4), indicating that photobleaching requires the presence of Carbo-4NP. These observations suggest that an oxidative quenching pathway operates in the absence of an external reductant, ultimately resulting in irreversible photobleaching of RB. To enable the completion of the photocatalytic cycle, NaAsc (25–400 µM) was added to solutions containing Carbo-4NP (50 µM) and RB (5 µM), which was found to minimise photobleaching in a dose-dependent manner (Fig. S5). In the presence of NaAsc (400 µM), complete release of 4-nitroaniline was observed within 2 minutes, as evidenced by the appearance of the characteristic absorption band at 380 nm, while the RB absorption band remain unchanged (Fig. 1b), showing superior activation kinetics than previously reported direct UV light activation, which required hours.^15^ No reduction of Carbo-4NP was observed with NaAsc (400 µM) under green-light irradiation in the absence of RB, indicating that the observed reduction of Carbo-4NP is mediated by photoactivated RB (Fig. S6). HPLC analysis further confirmed the release of 4-nitroaniline (Fig. S7). Interestingly, under nitrogen, photoreduction proceeded significantly faster, suggesting that oxygen may compete with the Pt(iv) photoreduction pathway (Fig. S8).

**Fig. 1:**
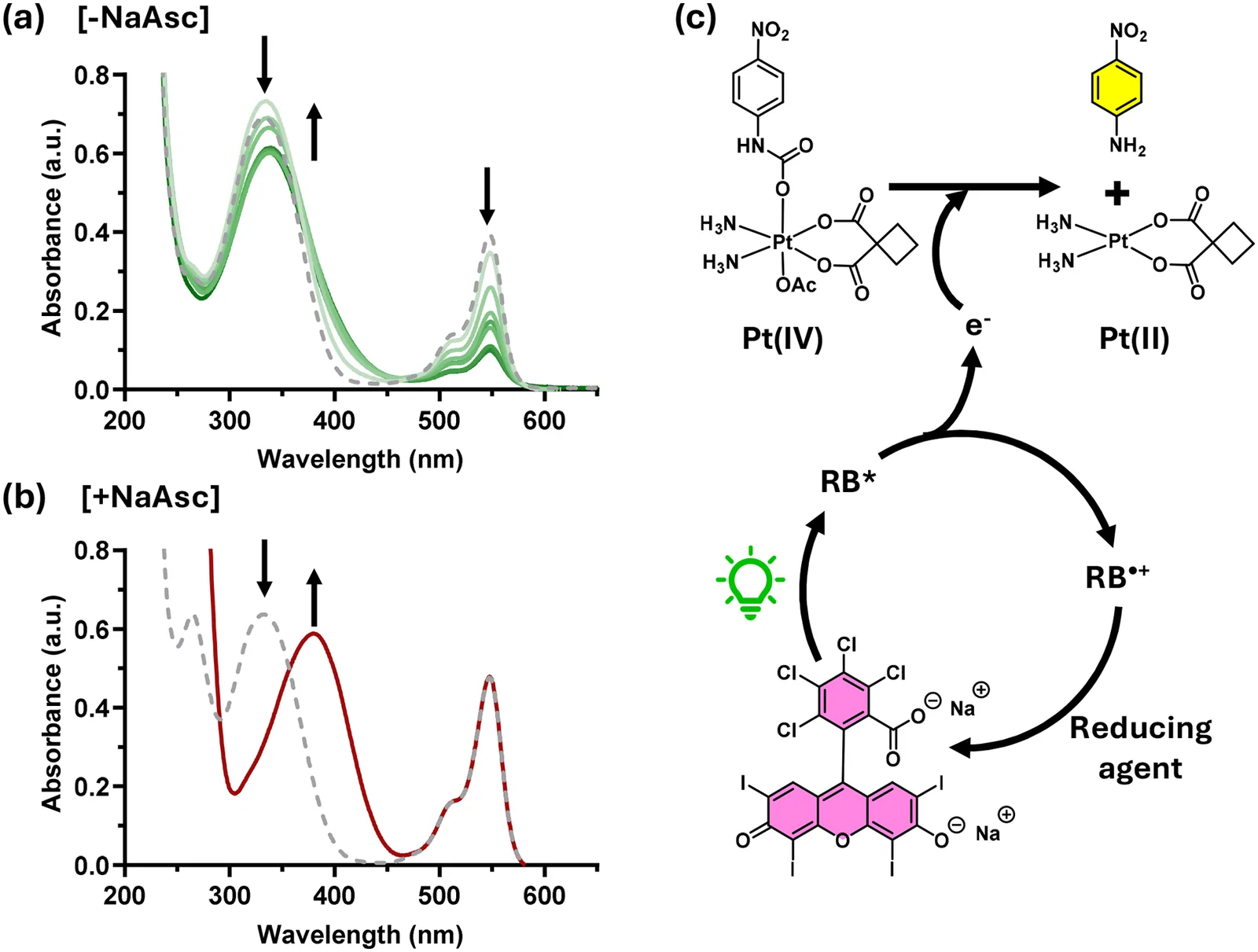
(a) Absorption spectra of Carbo-4NP (50 µM) containing RB (5 µM) irradiated every 15 s from 0 s (grey) to 120 s (dark green). (b) Absorption spectra of Carbo-4NP (50 µM) and RB (5 µM) in deionised water (DI) containing NaAsc (400 µM) before (grey dashed,) and after 2 min (red) green light irradiation. (c) The proposed reduction mechanism of Carbo-4NP using RB as photocatalyst. Green light forms an excited Rose Bengal species (RB*) that reduces Pt(iv) to Pt(ii) resulting in a cationic Rose Bengal (RB˙^+^). The cycle shows that photobleaching can be avoided by using a reducing agent allowing the reuse of the photocatalyst.

To further support the proposed photocatalytic cycle between RB, the reductant (NaAsc) and Carbo-4NP (Fig. 1c), additional biologically relevant reductants, including NADH and Fe(ii), were investigated and likewise found to maintain the RB absorption band (Fig. S9). In addition, when performed in HEPES buffer (10 mM, pH = 7.40), no reductant was required for photoconversion of Carbo-4NP, consistent with the ability of the tertiary amine functionality of HEPES to act as a sacrificial electron donor (Fig. S10).^24^ Having established the photocatalytic cycle, we next sought to determine the minimum amount of RB required for the complete conversion of Carbo-4NP to 4-nitroaniline. Remarkably, RB concentrations as low as 40 nM were sufficient at 2 min light irradiation (Fig. S11).

Structural analogues of RB, namely Eosin Y and fluorescein, were investigated to examine the influence of xanthene halogenation on the photocatalytic reduction of Pt(iv) complexes. Eosin Y (5 µM) and Carbo-4NP (50 µM) were irradiated with green light (520 nm) in the presence of NaAsc (400 µM) for 2 min and analysed by UV-Vis spectroscopy and HPLC (Fig. S12). Compared with RB, Eosin Y required slightly longer irradiation times to achieve complete release of 4-nitroaniline from Carbo-4NP (Fig. S12). In contrast, the non-halogenated analogue fluorescein (5 µM) produced less-substantial spectral changes following irradiation with Carbo-4NP (50 µM) and NaAsc (400 µM), indicating slower Pt(iv) reduction (Fig. S13 and S14). These results are consistent with the heavy atom effect, whereby extensive halogenation enhances spin–orbit coupling, promotes intersystem crossing, and increases triplet-state population, resulting in more efficient photocatalytic reduction.^25^

To further investigate the redox processes underlying the proposed photocatalytic cycle, a cyclic voltammetry (CV) study was performed for all molecular components. RB (1 mM) displayed an irreversible cyclic voltammogram with a pronounced cathodic peak (*E*_p,c_) at −0.90 V *vs.* Ag/AgCl (Fig. S12). With increasing concentrations of Carbo-4NP (*E*_p,c_ = −0.46 V, Fig. S15), the cathodic peak of RB (*E*_p,c_ = −0.93 V) was found to increase in current (Fig. S16). The increase in cathodic current is consistent with an electrochemically mediated process,^26^ whereby RB behaves as a chemical mediator for the reduction of Carbo-4NP. To further verify this observation, we applied −0.95 V to an aqueous solution of Carbo-4NP in the presence and absence of RB. UV-Vis analysis showed no detectable change in absorption without RB. In contrast, we observed a clear increase in absorption associated with the 4-nitroaniline peak when RB was present. These results support RB's role as a chemical mediator in facilitating Pt(iv) reduction in Carbo-4NP at an applied potential of −0.95 V (Fig. S17). Similar to RB, Eosin Y and Fluorescein showed a cathodic peak at −0.98 and −0.94 V, respectively (Fig. S18). However, the degree to which the cathodic current of Eosin Y increased was found to be a lesser extent compared to RB, and Fluorescein showed minimal changes to the cathodic current in the presence of Carbo-4NP (Fig. S19). To mitigate potential interference from the 4-nitro functionality of Carbo-4NP (Fig. S20), we next investigated whether similar behaviour was observed with the Pt(iv) complex (Scheme 1), CarboPhen^14^ (*E*_p,c_ = −0.70 V, Fig. S21) and the fluorogenic Pt(iv) complex, CarboNap (*E*_p,c_ = −0.23 V, Fig. S22). Comparable to Carbo-4NP, both CarboPhen and CarboNap produced pronounced current enhancement with RB, whereas only negligible changes were observed with Eosin Y and fluorescein (Fig. S23 and S24). Complete reduction of CarboPhen and CarboNap using RB as the photocatalyst under green-light irradiation was confirmed by HPLC, which confirmed electrochemical findings (Fig. S25). Together, these electrochemical data complement the photophysical studies, indicating that RB is uniquely effective at mediating electron transfer to carboplatin-derived Pt(iv) complexes.

Although Carbo-4NP has previously been shown to undergo direct photoreduction and, in this work, efficient RB-mediated photocatalytic reduction at nanomolar catalyst concentrations, colorimetric reporters are not suitable for monitoring Pt(iv) reduction in living cells. We therefore next sought to evaluate the photocatalytic potential of RB using our previously reported fluorogenic Pt(iv) prodrug, CarboNap. As shown in Fig. 2a, green-light irradiation of a solution containing CarboNap (5 µM), RB (0.5 µM), and NaAsc (40 µM) resulted in a time-dependent increase in fluorescence emission intensity. In contrast, only minimal fluorescence enhancement was observed in the absence of RB under identical irradiation conditions (Fig. S26). As previously reported,^6^ direct irradiation of CarboNap with blue light (415 nm, 27.2 mW cm^−2^) produced a significant increase in fluorescence emission both in the presence and absence of NaAsc (Fig. 2b and c). Comparison of the activation kinetics showed that direct blue-light irradiation resulted in more rapid release of Nap-NH_2_, whereas RB-mediated indirect activation required the presence of a sacrificial reductant to achieve a near-comparable rate of activation (Fig. 2c). HPLC analysis confirmed the release of the fluorescent fluorophore, Nap-NH_2_ following both direct and indirect activation (Fig. S25 and S27). With both activation strategies proving effective, we therefore next investigated whether this photocatalytic strategy could be translated to living cells.

**Fig. 2:**
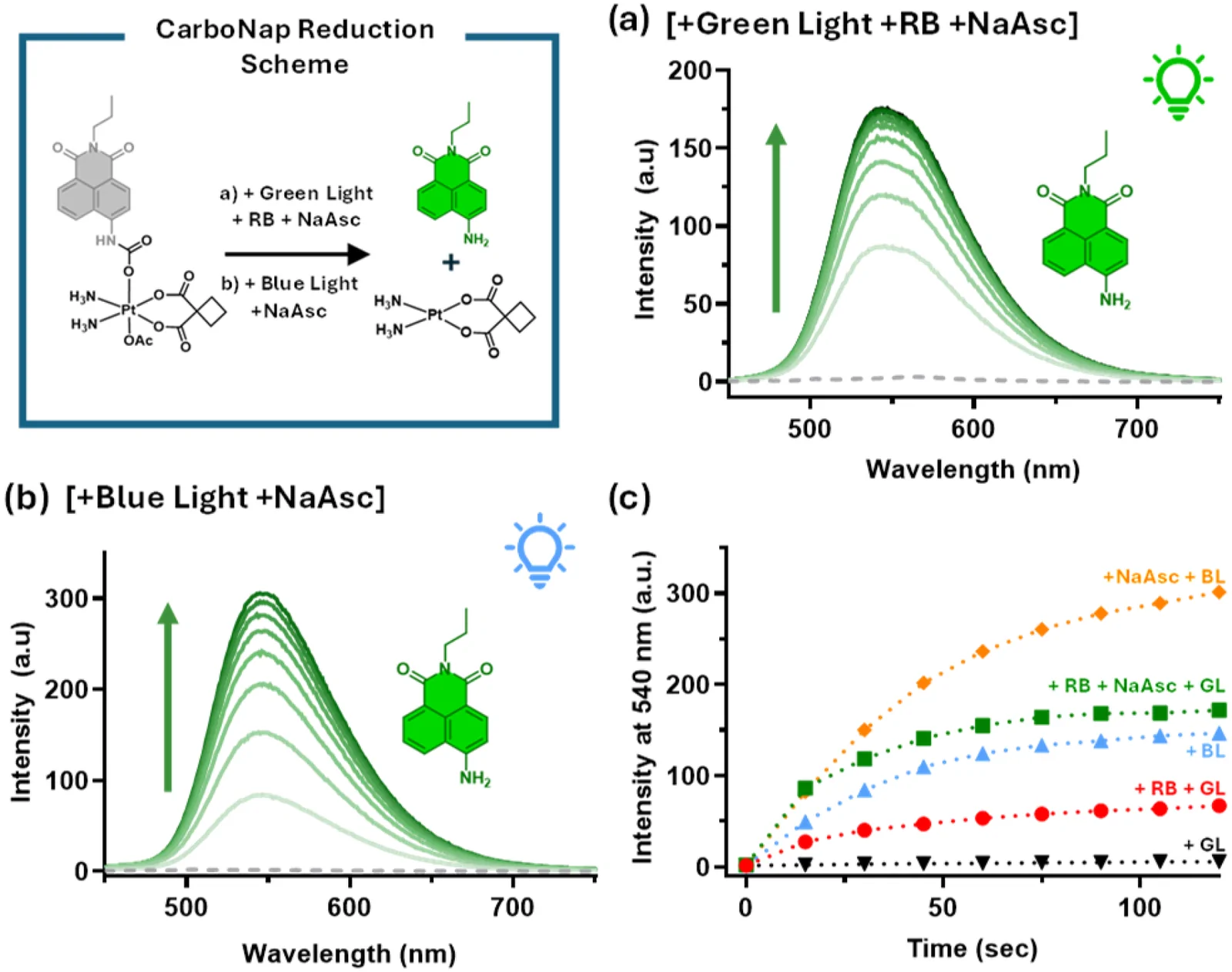
(a) Fluorescence emission spectra of CarboNap (5 µM) incubated with RB (0.5 µM) and NaAsc (40 µM) after being irradiated with green light from 0 (grey) to 120 s (dark green). Excitation: 430, emission: 450–800 nm, Exc slit: 5 nm, Em. slit: 5 nm. (b) Fluorescence emission spectra of CarboNap (5 µM) incubated with NaAsc (40 µM) after being irradiated with blue light from 0 (grey) to 120 s (dark green). Excitation: 430, emission: 450–800 nm, Ex. slit: 5 nm, Em. slit: 5 nm. (c) Fluorescent Intensity at 540 nm against light irradiation time (s) for CarboNap irradiated using blue light without (blue) and with NaAsc (orange), CarboNap irradiated using green light (black), CarboNap in presence of RB and using green light (red) and CarboNap in presence of RB and NaAsc using green light irradiation (green).

As the incubation time required for efficient intracellular accumulation of Pt(iv) prodrugs remains poorly defined, head and neck squamous cell carcinoma (HN5) cells were first incubated with CarboNap (10 μM) for varying durations (2, 4, 8 and 16 h) followed by direct light irradiation (415 nm, 27.2 mW cm^−2^) (Fig. 3 and Fig. S28). Direct photoactivation was used as a rapid means of assessing the intracellular accumulation of CarboNap *via* monitoring changes in fluorescence emission intensity upon blue light irradiation. Following 16 h incubation, irradiation with blue light for approximately 10 min produced a significant increase in intracellular fluorescence, as shown in Fig. 3b by fluorescence microscopy (Fig. 3a, b and Fig. S28). In contrast, shorter incubation periods (2 h and 4 h) produced little to no fluorescence activation; a minimum incubation time of 8 h was required to achieve sufficient intracellular accumulation for detectable photoactivation (Fig. S28). We next determined the minimum irradiation time required to induce a maximum change in fluorescence intensity (Fig. 3c and Fig. S29). Irradiation followed by measuring fluorescence intensity, showed there to be a time-dependent increase in fluorescence intensity, with maximal activation achieved after 8 min of blue-light irradiation (Fig. 3c).

**Fig. 3:**
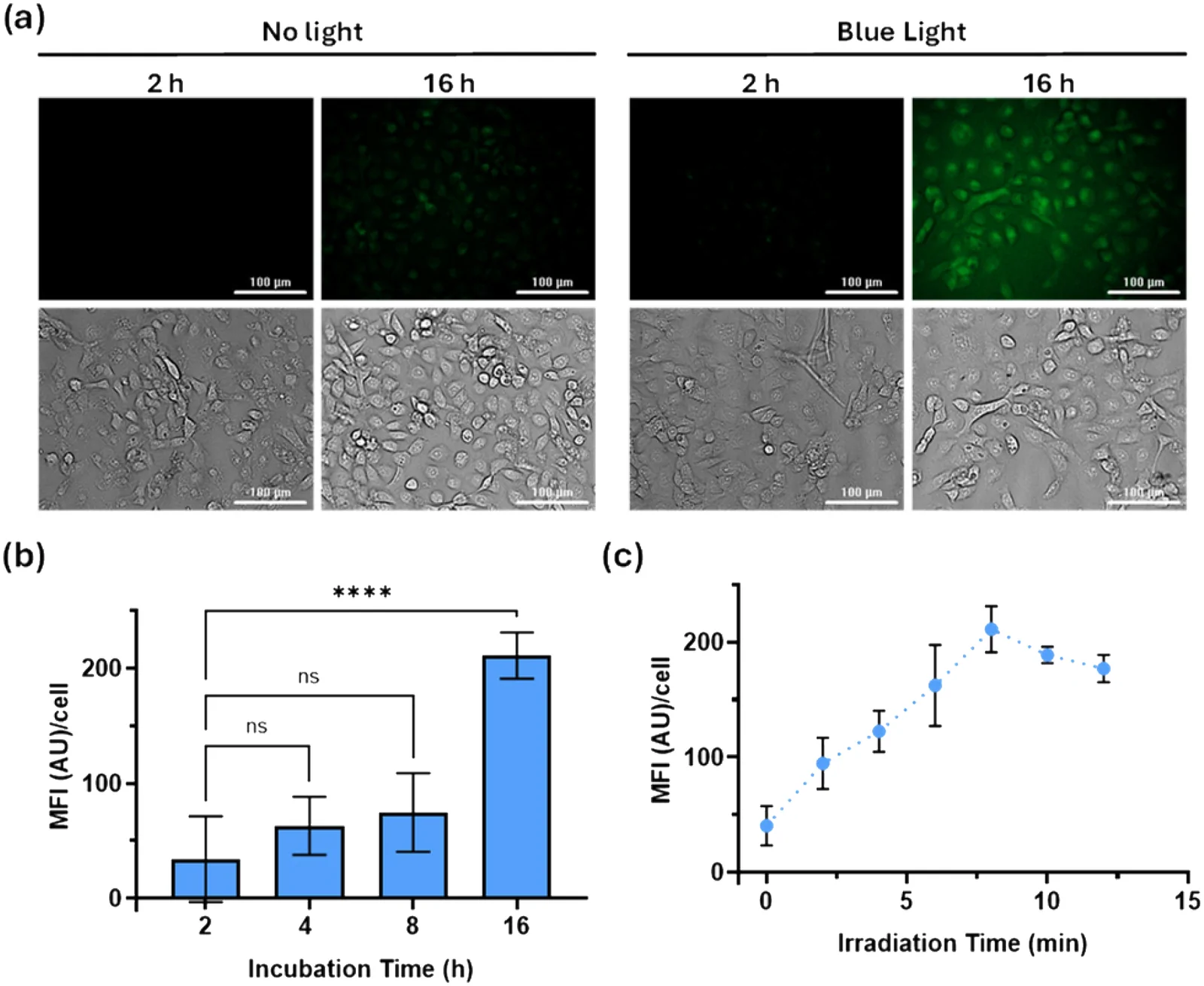
(a) Fluorescent images of HN5 cells incubated with CarboNap (10 µM) for 2 and 16 h before and after blue light (415 nm, 27.2 mW cm^−2^) irradiation for 8 min, with representative pictures shown (scale bar = 100 µm). (b) Fluorescence intensity of HN5 cells incubated with CarboNap (10 µM) for 2, 4, 8 and 16 h and after blue light (415 nm, 27.2 mW cm^−2^) irradiation for 8 min. (c) Irradiation time-dependent fluorescence intensity of HN5 cells incubated with CarboNap (10 µM) for 16 h. Each picture was quantified using Fiji. Data shown in b is presented as mean of 10 individual cells + SD. Statistical testing was done using one-way ANOVAs comparing to 2 h, where ns = non-significant, *p < 0.0332, **p < 0.0021, ***p < 0.0003, ****p < 0.0001.

With the optimal incubation and irradiation time established, we next wanted to determine the efficiency of indirect light-mediated activation of CarboNap with RB in HN5 cells. Green-light irradiation in the absence of RB resulted in little to no change in fluorescence intensity emission intensity, whereas in the presence of RB, a 2.5-fold enhancement was seen (Fig. 4a and Fig. S30). Attempts were made to further optimise intracellular photocatalytic activation between RB and CarboNap: CarboNap (10 μM) was exposed to increasing concentrations of RB (0.02–12.5 μM) and irradiated with green light (520 nm, 25.1 mW cm^−2^) for 8 min (Fig. S31). Quantification of fluorescence intensity showed that a minimum RB concentration of 12.5 μM was required to produce a statistically significant increase in fluorescence compared to RB alone (Fig. 4c and Fig. S31). Control experiments confirmed that treatment with RB only (12.5 μM) produced negligible cellular fluorescence, demonstrating that the observed signal originated from the release of Nap-NH_2_ from CarboNap (Fig. S32). Strikingly, direct blue-light activation produced a ∼370-fold increase in normalised fluorescence intensity (Fig. 4b). Thus, RB-mediated photocatalysis was markedly less efficient than direct blue-light activation in cells. This reduced efficiency for indirect activation could be due to differences in localisation of CarboNap, RB and reductant or poor cellular uptake of RB. To test the latter hypothesis, a more cell-permeable derivative Rose Bengal diacetate^27^ (20 μM) was synthesised and evaluated (SI Synthetic Procedures, Fig. S38). No significant differences in fluorescence activation were observed relative to RB (20 μM) (Fig. S33), indicating that RB permeability is not the limiting factor for intracellular photocatalytic activation.

**Fig. 4:**
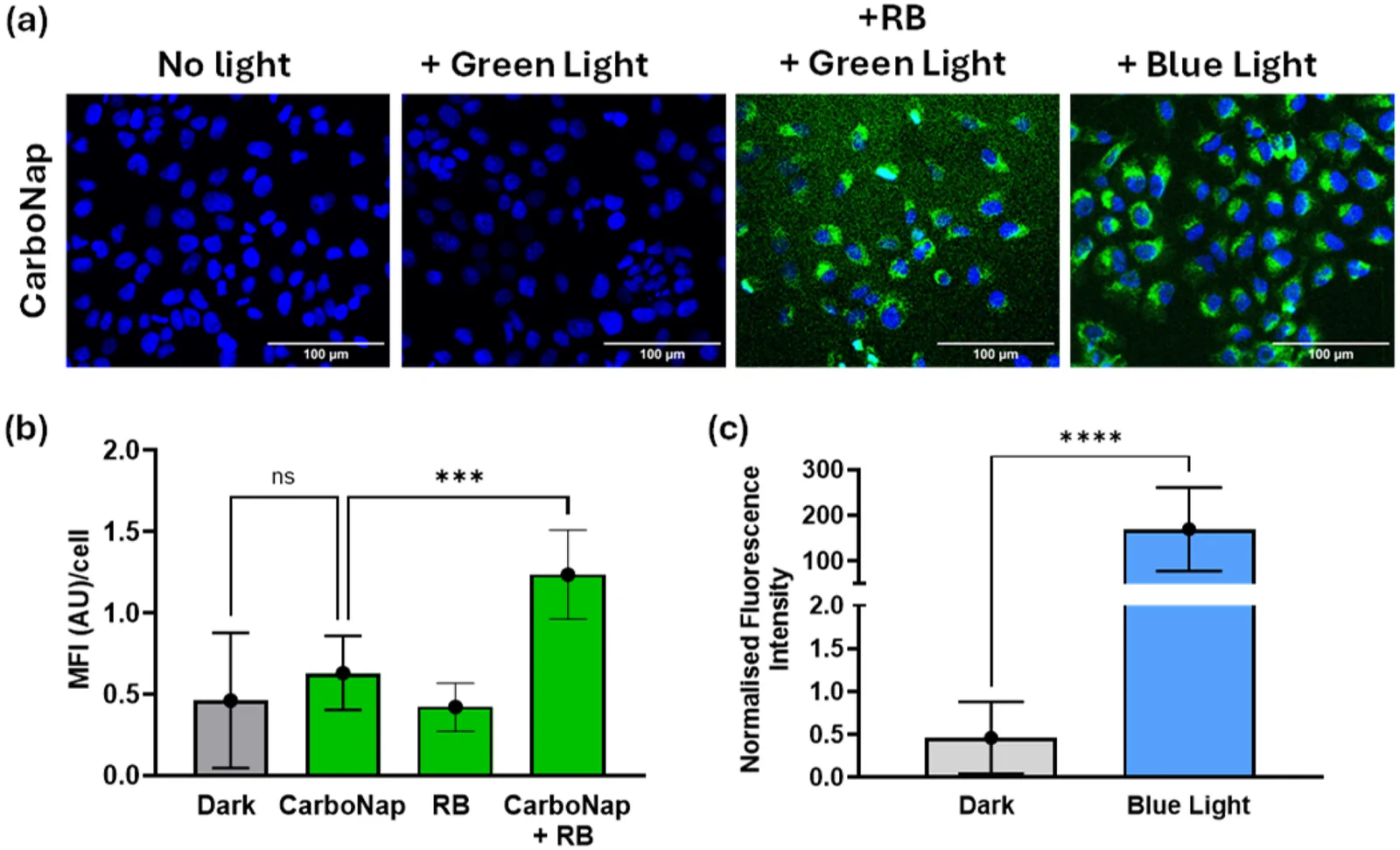
(a) Confocal images of HN5 treated with CarboNap (10 µM) with or without Rose Bengal (12.5 µM) and incubated overnight at 37 °C and exposed to blue (415 nm, 27.2 mW cm^−2^) or green light (520 nm, 25.1 mW cm^−2^) for 8 min before analysis by SoRa confocal, with representative pictures shown (scale bar = 100 µm). (b) Fluorescence intensity of HN5 cell containing CarboNap (10 µM) after being incubated with and without RB (12.5 µM) before (grey) and after green light irradiation (green) for 8 min (520 nm, 25.1 mW cm^−2^). (c) Fluorescence intensity of HN5 cells containing CarboNap (10 µM) before (grey) and after blue light irradiation (blue) for 8 min (415 nm, 27.2 mW cm^−2^). Each picture was quantified using Fiji. Data shown in b and c are presented as mean of 10 individual cells + SD. Statistical testing was done using one-way ANOVAs, where ns = non-significant, *p < 0.0332, **p < 0.0021, ***p < 0.0003, ****p < 0.0001.

Cell viability studies were next performed to evaluate the effect of direct and indirect activation of CarboNap on the proliferation of HN5 cells (Fig. 5a). Control experiments confirmed that blue- and green-light irradiation alone did not affect cell viability (Fig. S34). Direct photoactivation of CarboNap with blue light produced a 10-fold increase in cytotoxicity when compared to dark controls, with IC_50_ values of 51 μM (dark) and 5 μM (light), respectively (Fig. 5a and Fig. S35). For indirect activation, as RB itself exhibits phototoxicity, its toxicity profile was first established. The dark and light IC_50_ values were determined as 117 μM and 11.6 μM, following 8 min of green-light irradiation (Fig. S36). To minimise the influence of RB on the obtained cytotoxicity from CarboNap, 2.5 μM of RB was selected for subsequent indirect activation experiments. Under these conditions, treatment of HN5 cells with CarboNap and RB followed by green-light irradiation produced a small enhancement in cytotoxicity when compared to cell viability without RB (Fig. 5b). Although the use of RB was found ineffective in cells, photocatalytic Pt(iv) activation may prove advantageous in applications where photocatalyst localisation can be more tightly controlled in nanoparticle formulations to locally deliver cytotoxic Pt(ii).^17–19,28^

**Fig. 5:**
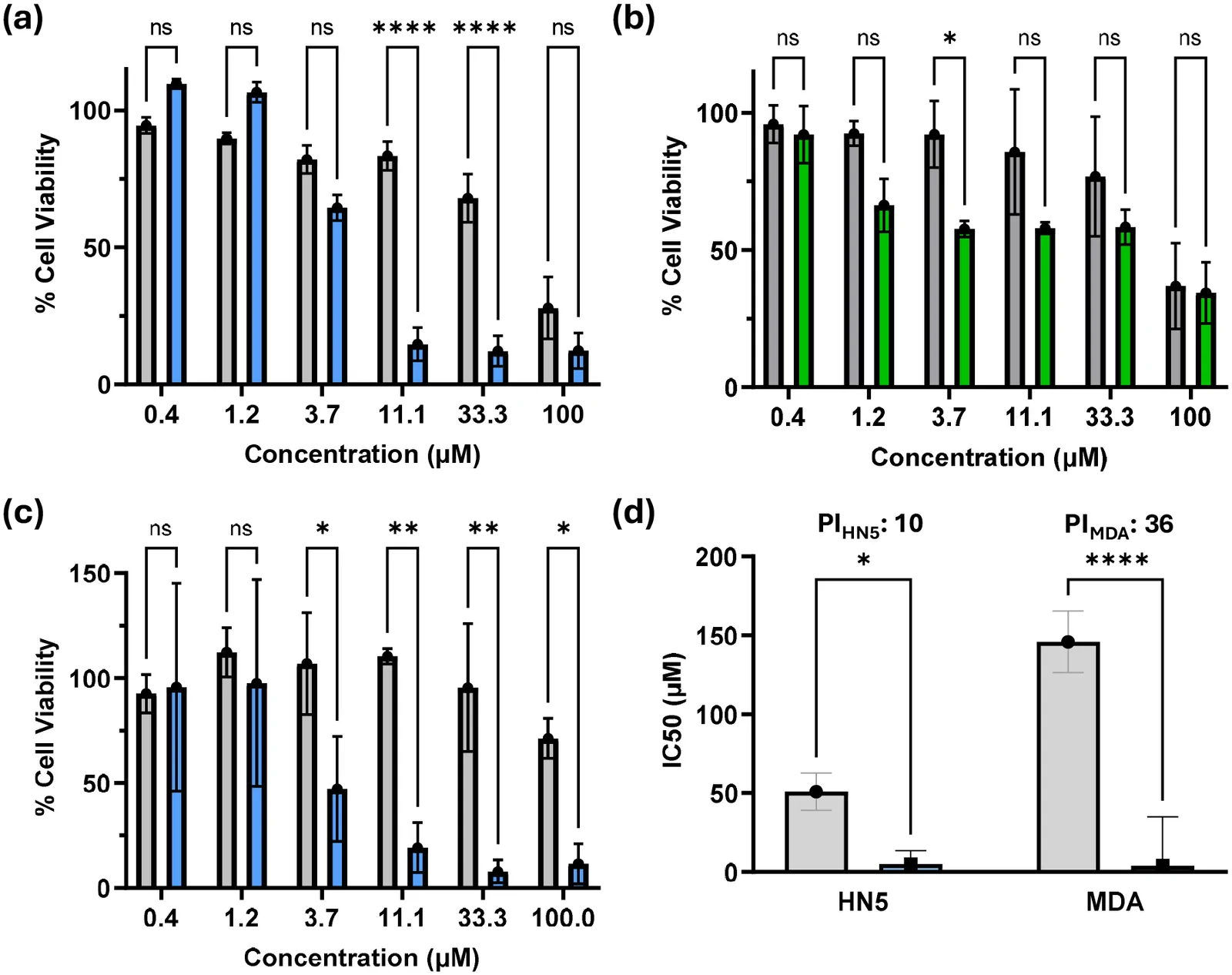
(a) Cell cytotoxicity of Human head and neck squamous carcinoma cells (HN5) incubated overnight with different concentrations of CarboNap, irradiated for 0 (grey) and 8 min (blue) with blue light (415 nm, 27.2 mW cm^−2^), and after incubating for an extra 24 h. (b) Cell cytotoxicity of HN5 cells co-incubated overnight in with (green) and without (grey) Rose Bengal (2.5 µM) and different concentrations of CarboNap, irradiated for 8 min with green light (520 nm, 25.1 mW cm^−2^), and after incubating for an extra 24 h. (c) Cell cytotoxicity of triple-negative breast cancer cells (MDA-MB-231) incubated overnight with different concentrations of CarboNap, irradiated for 0 (grey) and 8 min (blue) with blue light (415 nm, 27.2 mW cm^−2^), and after incubating for an extra 24 h. Data shown in a, b and d are *n* ≥ 3 (c is *n* ≥ 2), data is presented as mean + SEM. Statistical testing was done using two-way ANOVAs, where ns = non-significant, *p < 0.0332, **p < 0.0021, ***p < 0.0003, ****p < 0.0001. (d) Bar chart of CarboNap IC50 in HN5 and MDA cells after dark (grey) and blue light (blue) treatment. IC50 in HN5 is 50.9 μM after dark treatment, and 5.0 μM after blue light irradiation. IC50 in MDA is 108.9 μM after dark treatment, and 3.4 μM after blue light irradiation. The efficiency of blue light treatment in each cell is presented as phototoxicity index (PI).

To determine whether direct photoactivation was effective in other types of cells, CarboNap was evaluated in triple-negative breast cancer (MDA-MB-231) cells using the same irradiation protocol (Fig. 5c and Fig. S37). Blue-light irradiation significantly reduced cell viability from approximately 100% to 47% at 3.7 μM CarboNap and substantially lowered the IC_50_ compared with dark controls (Fig. 5c and d). The calculated phototoxicity index (PI)^29^ was higher in MDA-MB-231 cells (PI = 36) than in HN5 cells (PI = 10), demonstrating that direct photoactivation effectively enhances the cytotoxicity of CarboNap across two different cancer cell lines and represents a promising avenue for further exploration and chemical optimisation.

This work compared direct and photocatalyst-mediated activation of Pt(iv) complexes. Both approaches achieved rapid and complete reduction of Pt(iv) to Pt(ii) in solution. However, indirect activation required the presence of reducing agents, such as NaAsc, to avoid RB photobleaching and for complete reduction of Pt(iv) complexes. Thus, while both strategies proved effective in solution, direct activation proved to be a superior method, resulting in greater enhancement in intracellular fluorescence intensity and cytotoxicity. These findings establish direct light activation as a promising platform for the controlled activation of Pt(iv) prodrugs and underscore its potential to improve targeted platinum-based chemotherapy.

## Conflicts of interest

There are no conflicts to declare.

## Supplementary Material

DT-OLF-D6DT01745B-s001

## Data Availability

Data will be made available by the corresponding authors upon request. Supplementary information (SI) contains additional experimental data and methods. See DOI: https://doi.org/10.1039/d6dt01745b.
