# Supplementary material for: Comparative study of the direct and photocatalytic light activation of platinum(iv) prodrugs
Source: Dalton Trans. 2026 Oct 12. Online ahead of print. doi: 10.1039/d6dt01745b (PMC13678618; doi:10.1039/d6dt01745b)
Supplement: Supplementary file 1 [file DT-OLF-D6DT01745B-s001.pdf]

Supplementary Information (SI) for Dalton Transactions.
This journal is © The Royal Society of Chemistry

Supporting Information

Comparative study of the direct and photocatalytic light activation of platinum(IV) prodrugs

Kinjal Mileche,[a†] Susana M. Meza Huaman ,[a] Ke Wu,[a] Jake Vickery,[a] Hannah Coxhead,[a,b] Alexander N. Smith-Xu,[a] Patricia Juhl Allworthy,[a] Jovana Katrinka-Mavrak,[c] Aminesena Baser,[d] Safacan Kolemen,[d] Manuel M. Mueller,[a] Mahvash Tavassoli,[b] Ismael Diez-Perez,[a] Eleanor Stride,[c] John F. Callan,[e*] Adam C. Sedgwick[a*]

[a]*Department of Chemistry, King's College London, 7 Trinity Street, London, SE1 1DB, United Kingdom*

[b]*Centre for Host-Microbiome Interactions, King's College London, London, London, United Kingdom.*

[c]*Institute of Biomedical Engineering, Department of Engineering Sciences, Old Road Campus Research Building, University of Oxford, Headington, Oxford, UK.*

[d] *Department of Chemistry, Koç University, Istanbul 34450, Türkiye*

[e]*Biomedical Sciences Research Institute, University of Ulster, Coleraine, Northern Ireland, UK.*

Emails: j.callan@ulster.ac.uk and adam.sedgwick@kcl.ac.uk

Table of Contents

1. General Information and Methods S3

2. Synthetic Procedures S7

3. Additional Analyses S8

4. NMR Spectra S27

5. References S27

1. General Information and Methods

All chemicals and reagents were of analytical grade and purchased commercially. Fluorescence and absorbance spectra were collected on a Cary Eclipse Agilent Technologies spectrometer and Shimadzu UV-Vis spectrophotometer, respectively, using quartz cuvettes (1 cm path length). The photophysical studies were performed in deionised water, with the pH measured before and after green-light irradiation. The pH decreased by approximately 0.2 units following irradiation (from 7.02 to 6.79). NMR spectra were recorded on a 400 MHz Bruker Ascend equipped with a broadband probe and chemical shifts are reported as δ values in ppm. HPLC analyses was carried out using a Thermo Fisher Scientific Vanquish HPLC system with an Syncronis C-18 column [15 cm x 4.6 mm, 5 µm]; HPLC conditions: injection volume: 10 µL, flow rate: 1.5 mL/min, 25 °C, 0–35 min [5:95 MeCN:H_2O (both with 0.1% acetic acid), 5 min hold → 90:10 MeCN:H_2O with 0.1% acetic acid for 20 min, 5 min hold → 5:95 MeCN:H_2O (both with 0.1% acetic acid), 3 min → 5:95 MeCN:H_2O (both with 0.1% acetic acid), 2 min hold]. High Resolution Mass Spectrometry (HRMS) spectra were obtained using UPLC-Xevo G2 XS QToF HRMS spectrometers. For analytical work, all complexes investigated were dissolved in DMSO to prepare 10 mM stock solutions. Each stock solution was then diluted in deionised water. Fluorescence analysis used 5 µM working solutions of each Pt(IV) complex. A 70/30 ratio of milli-Q water/MeCN mixture was used to provide 50 µM working solutions of each probe for HPLC analysis. Light irradiation studies were performed 20 cm away from the light source in quartz cuvettes for the relevant time points with a blue (415 nm, 27.2 mW·cm^{-2}) and green (525 nm, 25.1 mW·cm^{-2}) Hooga Health light. The power irradiance at the sample plane was measured using a silicon photodiode power sensor (PH100-Si-OD1, gentec-eo) attached to a power meter (tuner, gentec-eo). Synthesis of Carbo-4NP, CarboNap and CarboPhen were previously reported.[1]

1.1. Electrochemical experiments

Electrochemical experiments were carried out using an Autolab IMP PGSTAT128N potentiostat (Metrohm, Netherlands) equipped with a three-electrode configuration comprised of a 3 mm glassy carbon working electrode, a platinum wire counter electrode, and a Ag/AgCl reference electrode. The working electrode was polished before each scan with alumina slurry (0.3 µM) and rinsed deionised water. Prior to each run, the electrolyte

solutions were deaerated with N_2. A total volume of 2 mL was used in each experiment. The basic cyclic voltammetry experiments had a concentration of 1 mM for each analyte, and the titration experiments consisted of 1 mM of Xanthene dye and varying concentrations of platinum (IV) prodrug (0 µM, 250 µM, 500 µM, 1000 µM).

1.2. Cell Culture

Head and neck squamous cell carcinoma HN5 (Prof. Mahvash Tavassoli, King's College London, UK) and triple-negative breast cancer MDA-MB-231 cells (purchased from ATCC) were cultured in DMEM supplemented with 10 % FBS, 1 % Penicillin-Streptomycin and 1 % Na-pyr in humified incubators at 37 ^oC with 5 % CO_2. Cells were periodically verified to be negative for mycoplasma using a Lonza MycoAlert® mycoplasma detection assay (LT07-318, Lonza). For experiments where light irradiation was used, cells were seeded in Gibco DMEM with GlutaMAX supplement (Gibco, A1896701) to circumvent absorption of light < 450 nm by phenol red.

1.7. MTT Assay

Blue light activation

Cells were seeded in 96 well plates (5000 cells/well) and allowed to adhere overnight. After treatment with CarboNap at the indicated concentrations for 24 h, the cells were exposed to blue light (415 nm, 20 cm, 19.3 mW) for 8 min. After an additional 24 h incubation, cells were carefully washed once with PBS and MTT reagent (0.8 mg/mL, Invitrogen) was added for 2h (37 ^oC) with the plate protected from light. MTT containing media was then removed and formazan crystals were solubilised with DMSO (100 µL, 30 min, 37 ^oC), protected from light. The absorbance was read at 620 nm (Hidex Sense). Data are shown as percentage viability relative to the untreated vehicle control.

Green light activation

Cells were seeded in 96 well plates (5000 cells/well) and allowed to adhere overnight. After treatment with CarboNap at the indicated concentrations with and without Rose Bengal (2.5 µM) for 24 h, the cells were exposed to green light (520 nm, 20 cm, 17.8 mW) for 8 min. After an additional 24 h, cells were carefully washed once with PBS and MTT reagent (0.8 mg/mL, Invitrogen) was added for 2h (37 ^oC) with the plate protected from light. MTT containing

media was then removed and formazan crystals were solubilised with DMSO (100 μL, 30 min, 37 °C), protected from light. The absorbance was read at 620 nm (Hidex Sense). Data are shown as percentage viability relative to the untreated vehicle control.

1.8. Fluorescent Microscopy

Cytation 5

For blue light activation, cells were seeded into 96-well plate and treated with CarboNap (10 μM) in DMEM media (supplemented with 10 % FBS, 1 % Penicillin-Streptomycin and 1 % Na-pyr) for 2, 4, 8 and 16 h before exposure to blue light (415 nm, 20 cm, 19.3 mW) or no exposure. Cells were washed three times with PBS, and media were added (Sigma Aldrich). Cells were visualised with Cytation 5, BioTek, and Gen5 software. Analysis of fluorescent images were conducted using Fiji. Fore green channel, 10 different cells of each picture were selected, and their mean intensity was measured maintaining the area constant. The area without cells was measured as background and subtracted from the measured mean intensity. Their average per cell yielded their corresponding mean fluorescence intensity (MFI).

Confocal imaging

For blue light activation, cells were seeded into 96-well plate and treated with CarboNap (10 μM) in DMEM media (supplemented with 10 % FBS, 1 % Penicillin-Streptomycin and 1 % Na-pyr) for 16 h before exposure to blue light (415 nm, 20 cm, 19.3 mW) for 8 min or no exposure. For green light activation, cells were seeded into 96-well plate and treated with CarboNap (10 μM) with and without Rose Bengal (0.02, 0.5, 2.5 and 12.5 μM) in DMEM media (supplemented with 10 % FBS, 1 % Penicillin-Streptomycin and 1 % Na-pyr). Cells were also treated only with Rose Bengal (12.5 μM) as control group. After 16 h incubation time, cells were exposed to green light (520 nm, 20 cm, 17.8 mW) for 8 min. Cells were washed three times with PBS, DMEM media and Hoechst were added (Sigma Aldrich). Cells were visualised with an Eclipse Ti-2 inverted microscope (Nikon Europe B.V.) with SoRa spinning disk super-resolution at 20x magnification with blue SoRa 1x and green SoRa 1x. Microscope settings were kept consistent between conditions with the following wavelengths used for each channel: blue excitation 405 nm, emission 430-475 nm; green excitation 488 nm, emission

499-551 nm. Analysis of non-overlapped confocal images were conducted using Fiji. For each channel, 10 different cells of each picture were selected, and their mean intensity for each channel was measured maintaining the area constant. The area without cells was measured as background and subtracted from the measured mean intensity. The mean intensity of green channel was normalised to the mean intensity of blue channel. Then, the normalised average per cell yielded their corresponding mean fluorescence intensity (MFI).

2. Synthetic Procedures

Diacetylated Rose Bengal

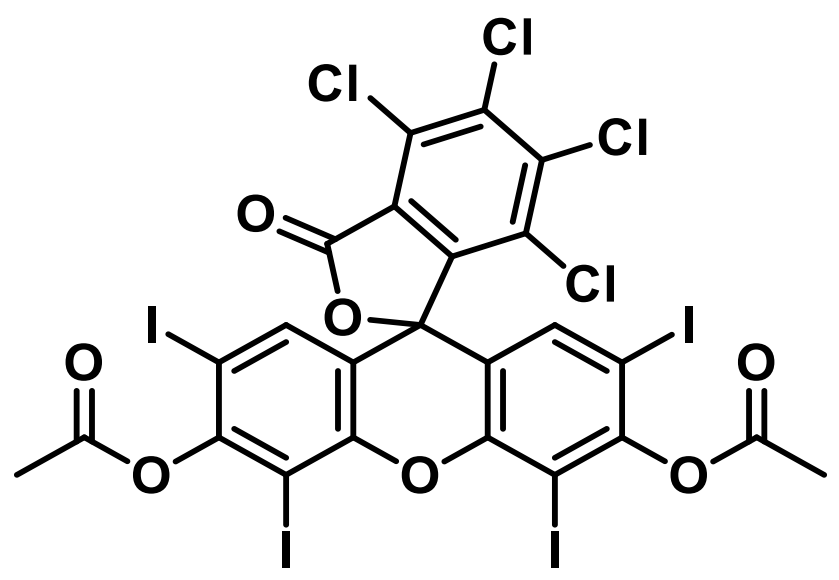

Diacetylated rose bengal was obtained following the reported method by Bottiroli *et al.*[2] Rose Bengal sodium salt (1 g, 0.98 mmol) was added to 50 mL of acetic anhydride stirred at room temperature for 20 h. The deep fuchsia colour of the solution slowly faded to a pale pink colour. The reaction mixture was then treated with a diluted aqueous solution of NaOH and extracted with ethyl acetate to afford the title compound as a pale pink solid. Yield (0.62 g, 60 %). ^{1}H NMR (400 MHz, DMSO-d_6) δ (ppm): 7.84 (s, 2H), 2.43 (s, 6H). NMR characterisation was in line with the previous literature report.[2]

3. Additional Analyses

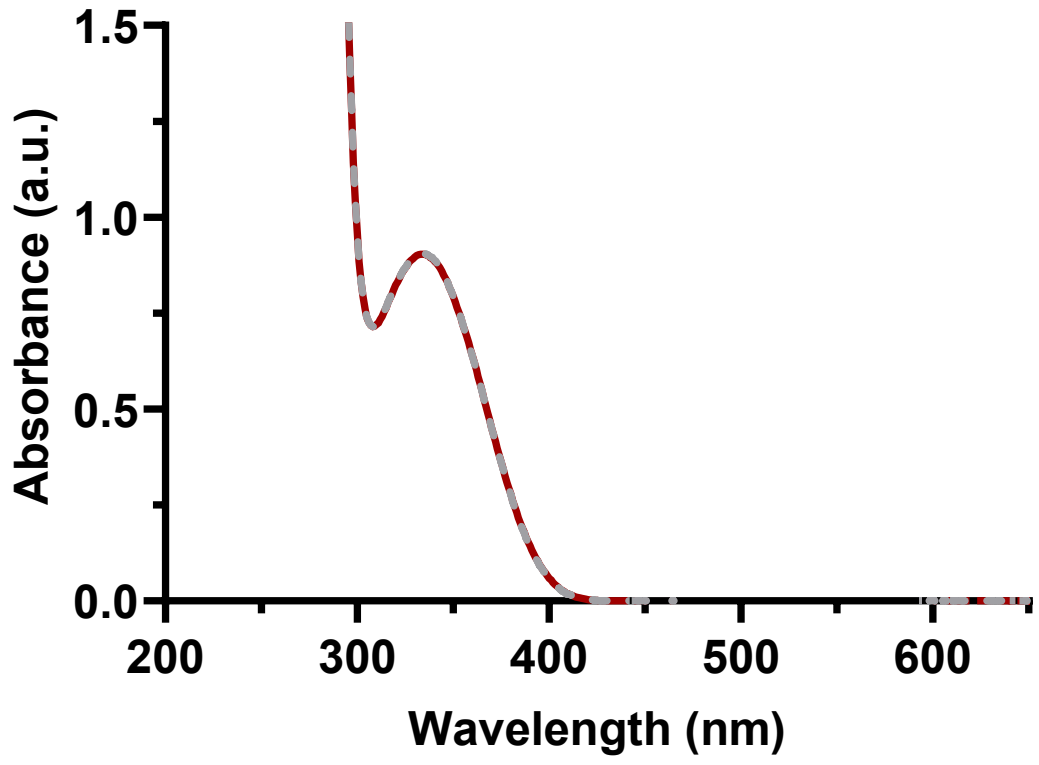

Figure S1. Absorption spectra of Carbo-4NP (50 µM) containing 1 mM NaAsc before (grey dashed) and after 40 min incubation (red) in deionised water.

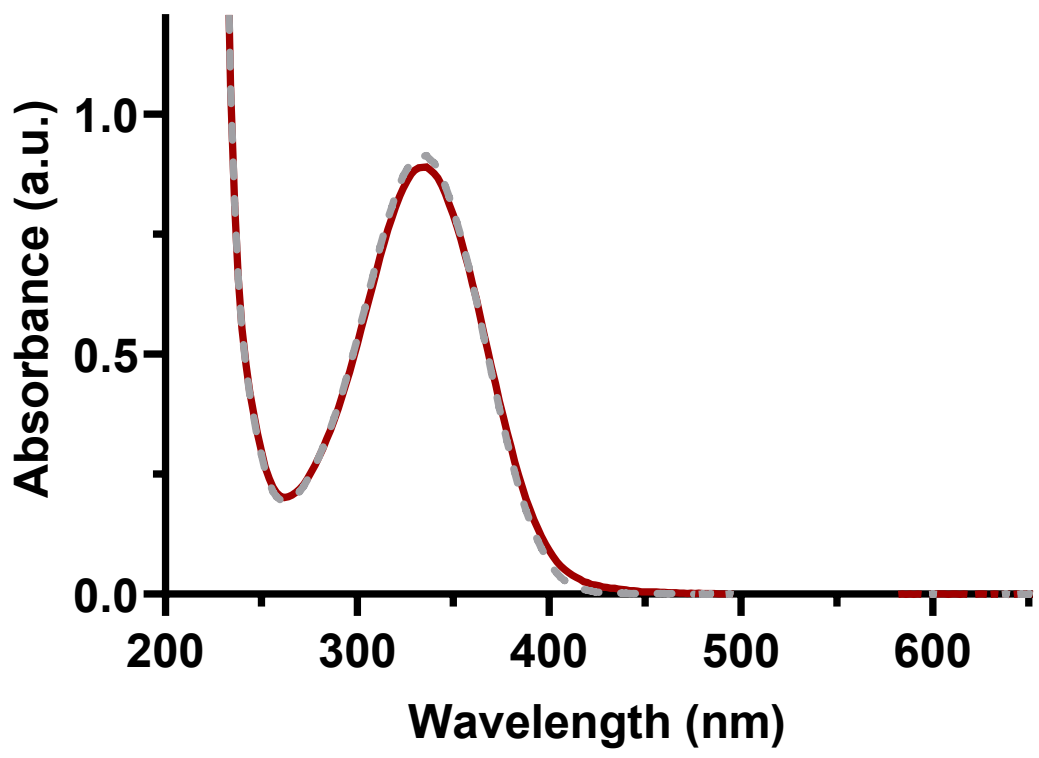

Figure S2. Absorption spectra of Carbo-4NP (50 µM) in DI water before (grey dashed) and after 40 min green light irradiation (red).

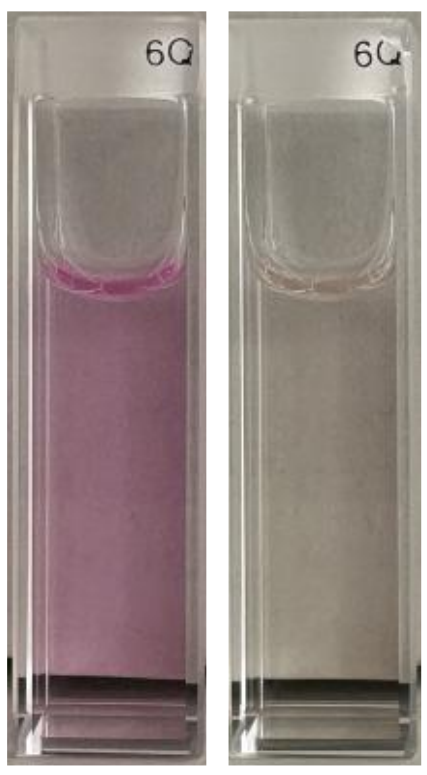

Figure S3. Picture of working solution containing Carbo-4NP (50 µM) and Rose Bengal (5 µM) in DI water before (left) and after (right) green light irradiation for 2 min.

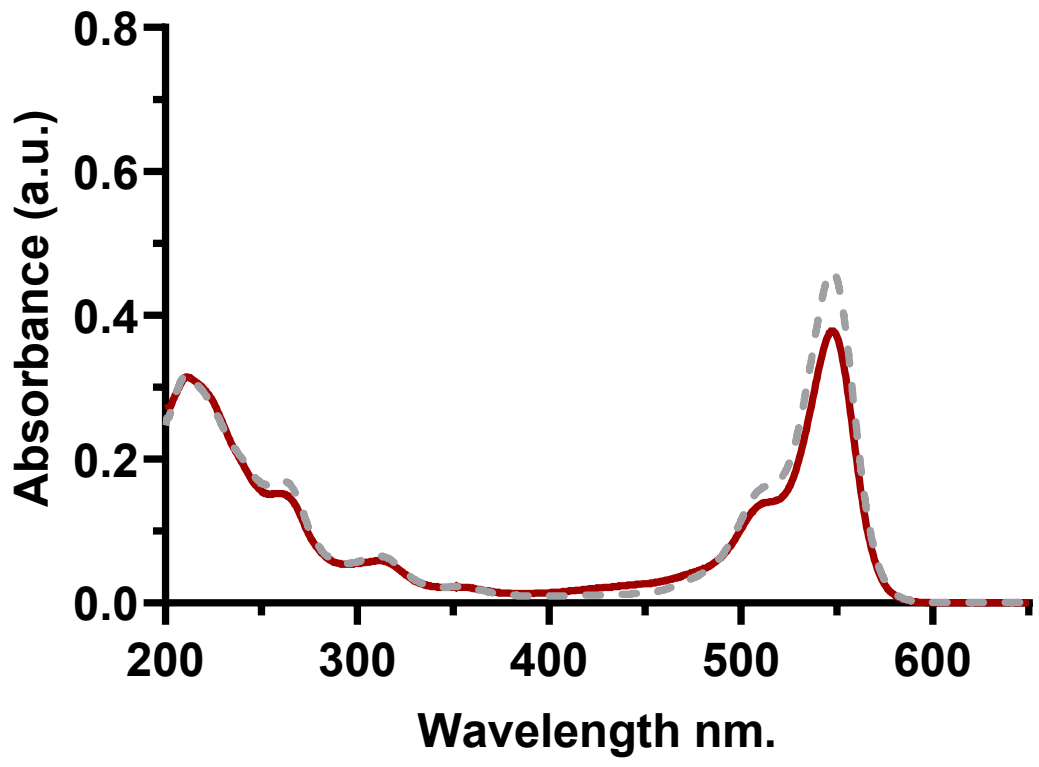

Figure S4. Absorption spectra of Rose Bengal (5 µM) in DI water before (grey dashed) and after 2 min green light irradiation (red).

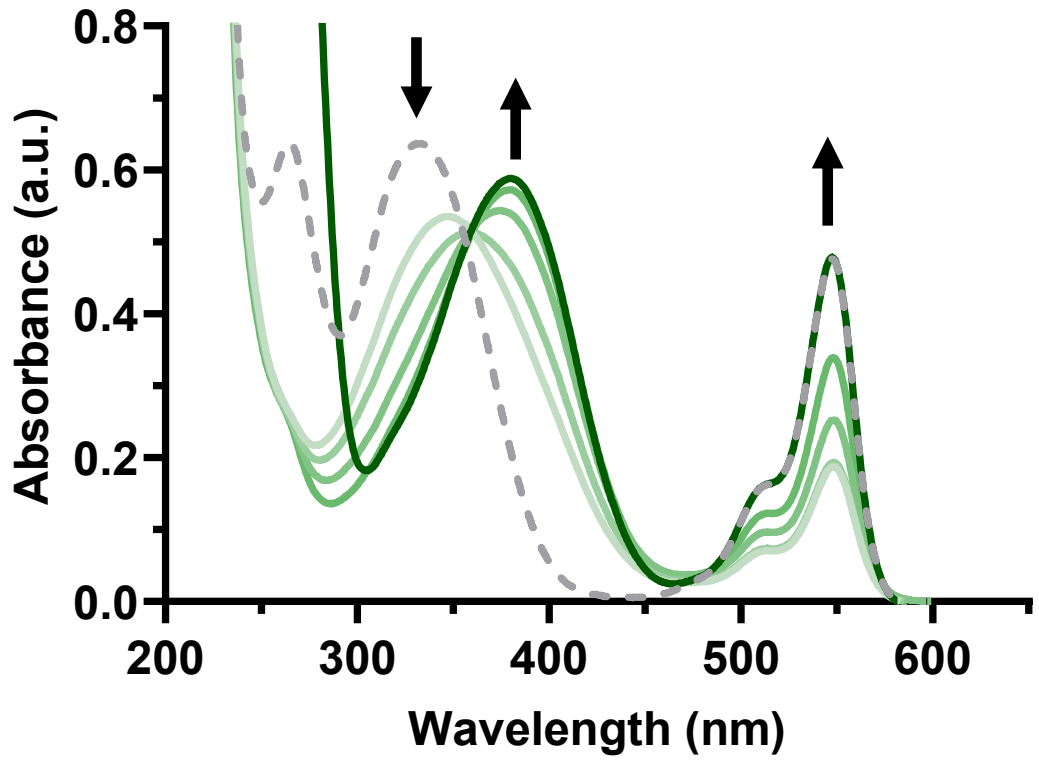

Figure S5. Absorption spectra of Carbo-4NP (50 µM) containing Rose Bengal (5 µM) before green light irradiation (grey dashed) and after 2 min light irradiation in the presence of different concentrations of NaAsc, ranging from 25 µM (light green) to 400 µM (dark green).

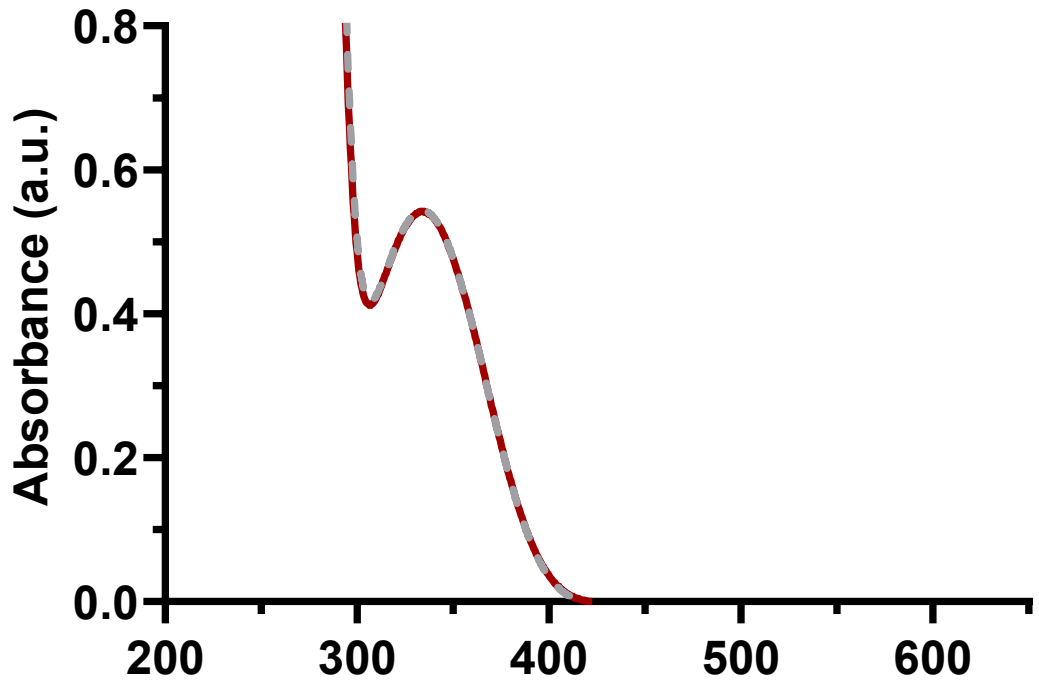

Figure S6. Absorption spectra of Carbo-4NP (50 µM) containing 400 µM NaAsc before (grey dashed) and after 2 min green light irradiation (red) in deionised water.

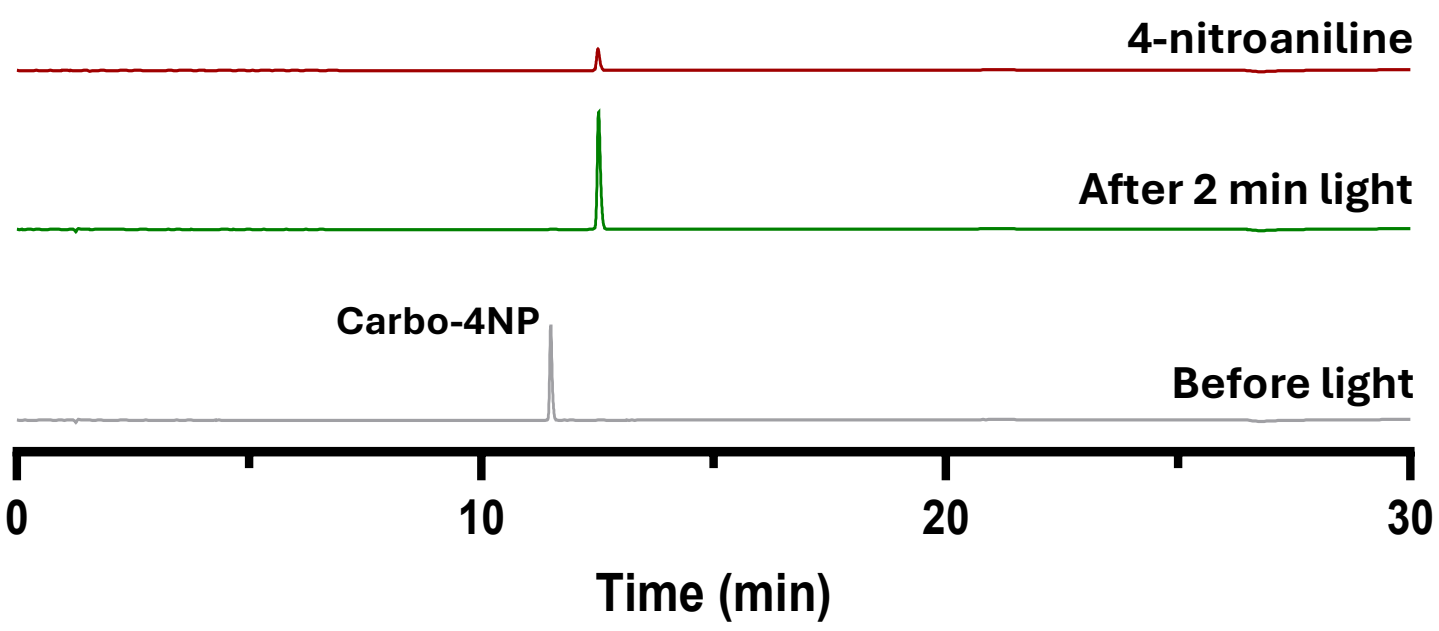

Figure S7. HPLC traces of Carbo-4NP (300 µM) in water containing Rose Bengal (30 µM) and NaAsc (2.4 mM) before (grey) and after 2 min green light irradiation (green) (absorption at 320 nm).

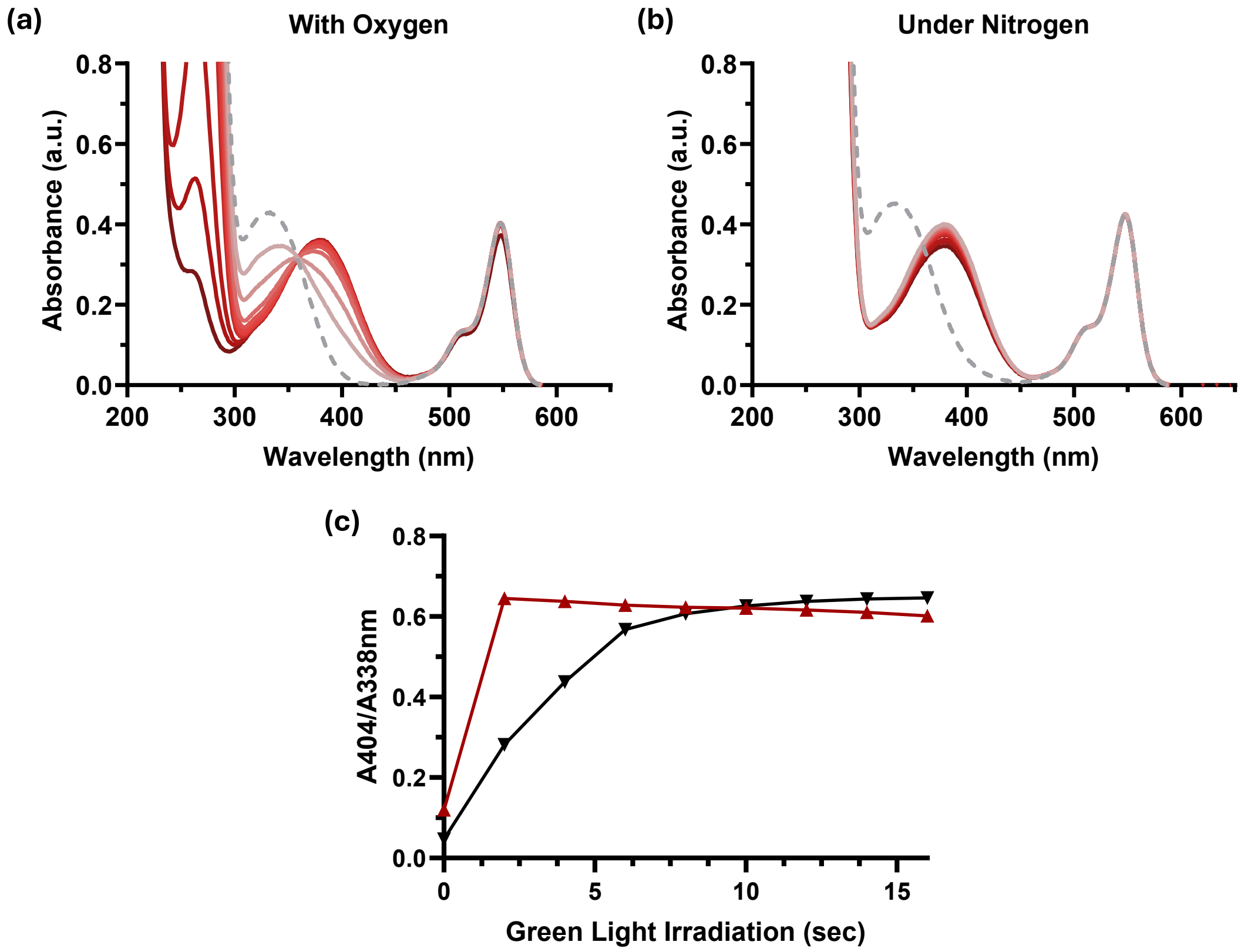

Figure S8. Change in absorption spectra of Carbo-4NP (50 µM) with NaAsc (400 µM) after green light irradiation for 0 to 60 sec (grey to dark red) in deionised water using Rose Bengal as photocatalyst (5 µM) in (a) the presence of oxygen and (b) under nitrogen. Samples were degassed under nitrogen for 2 min. (c) Change in absorption (A404/A338) of Carbo-4NP as a function of green light irradiation time in the presence (black) and the absence (red) of oxygen.

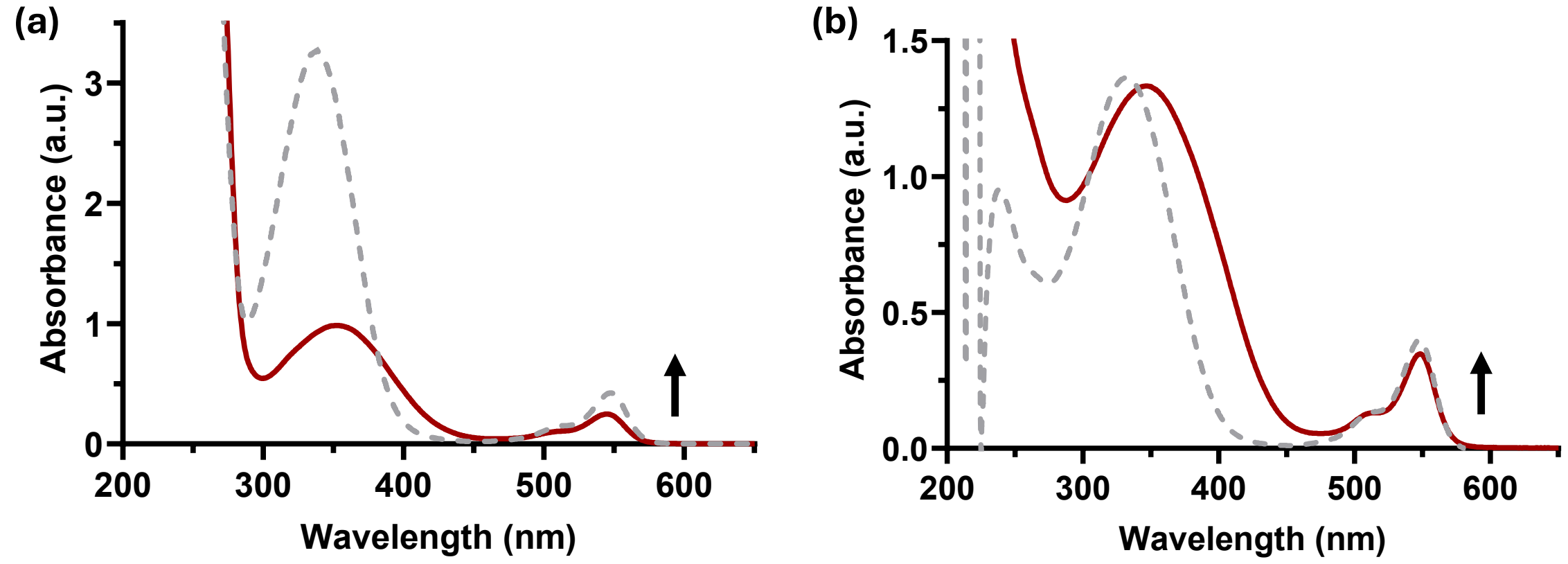

Figure S9. Absorption spectra of Carbo-4NP (50 μM) and Rose Bengal (5 μM) in DI water containing (a) NADH (400 μM) and (b) Fe(II) gluconate before (grey dashed) and after 2 min (red) green light irradiation.

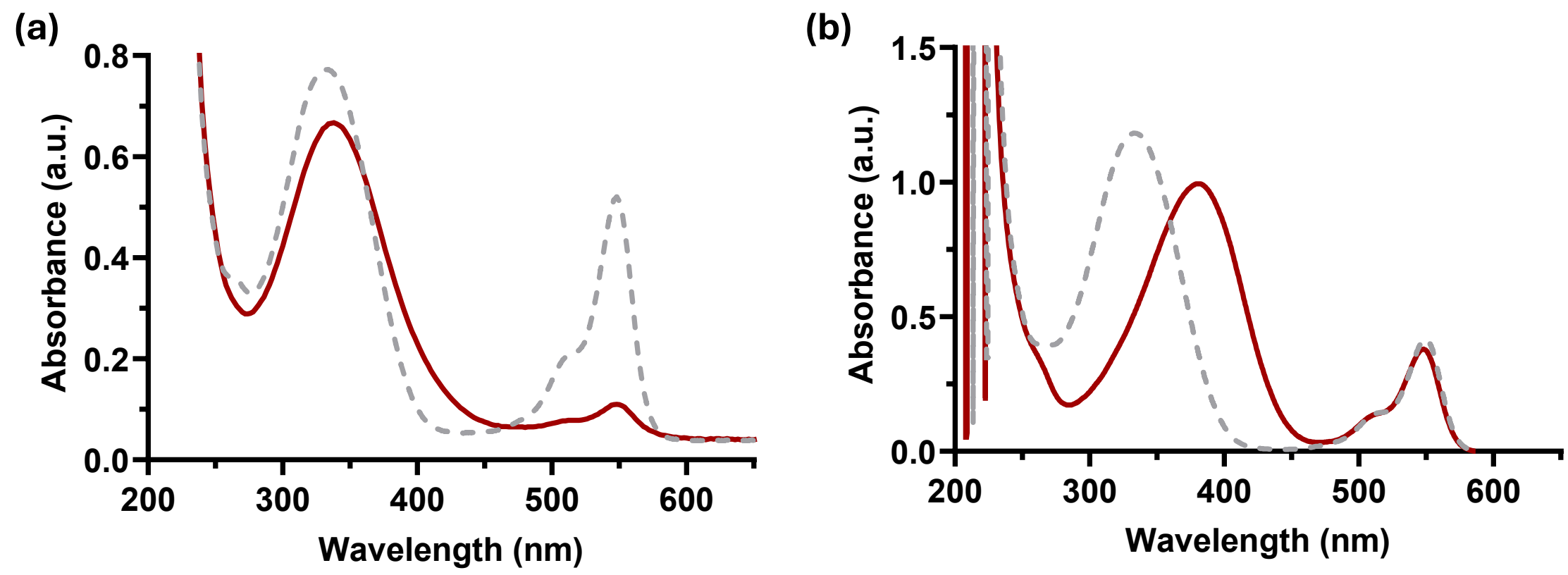

Figure S10. (a) Absorption spectra of Carbo-4NP (50 μM) containing Rose Bengal (5 μM) in PBS buffer solution (pH = 7.40) before (grey dashed) and after 2 min light irradiation (red). (b) Absorption spectra of Carbo-4NP (50 μM) containing Rose Bengal (5 μM) in HEPES buffer solution (pH = 7.40) before (grey dashed) and after 2 min light irradiation (red).

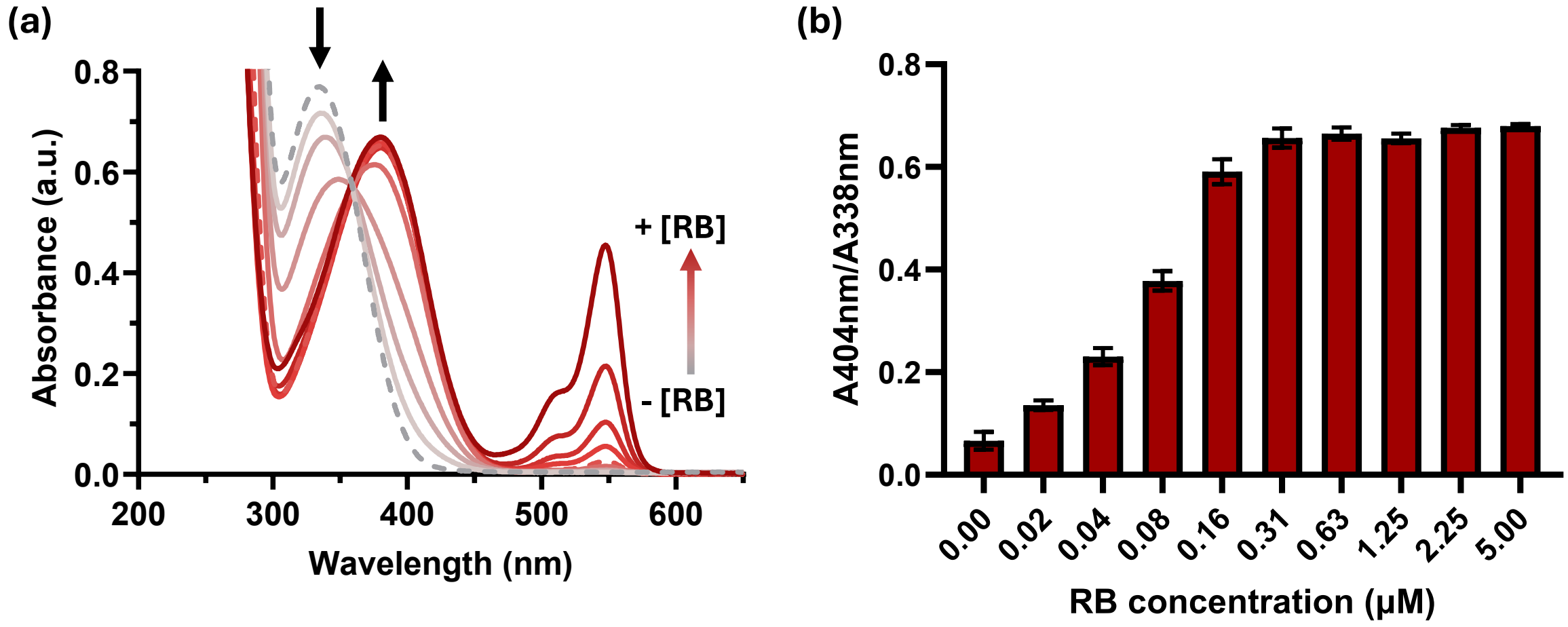

Figure S11. (a) Absorption spectra of Carbo-4NP (50 μM) containing NaAsc (400 μM) and Rose Bengal (5 μM) in DI water decreasing the concentration of Rose Bengal from 0 μM to 5 μM and irradiating for 2 min with green light. (b) Bar chart of the change in absorption (A404/A338) of Carbo-4NP with different concentration of RB after 2 min green light irradiation. Error bars represent SD.

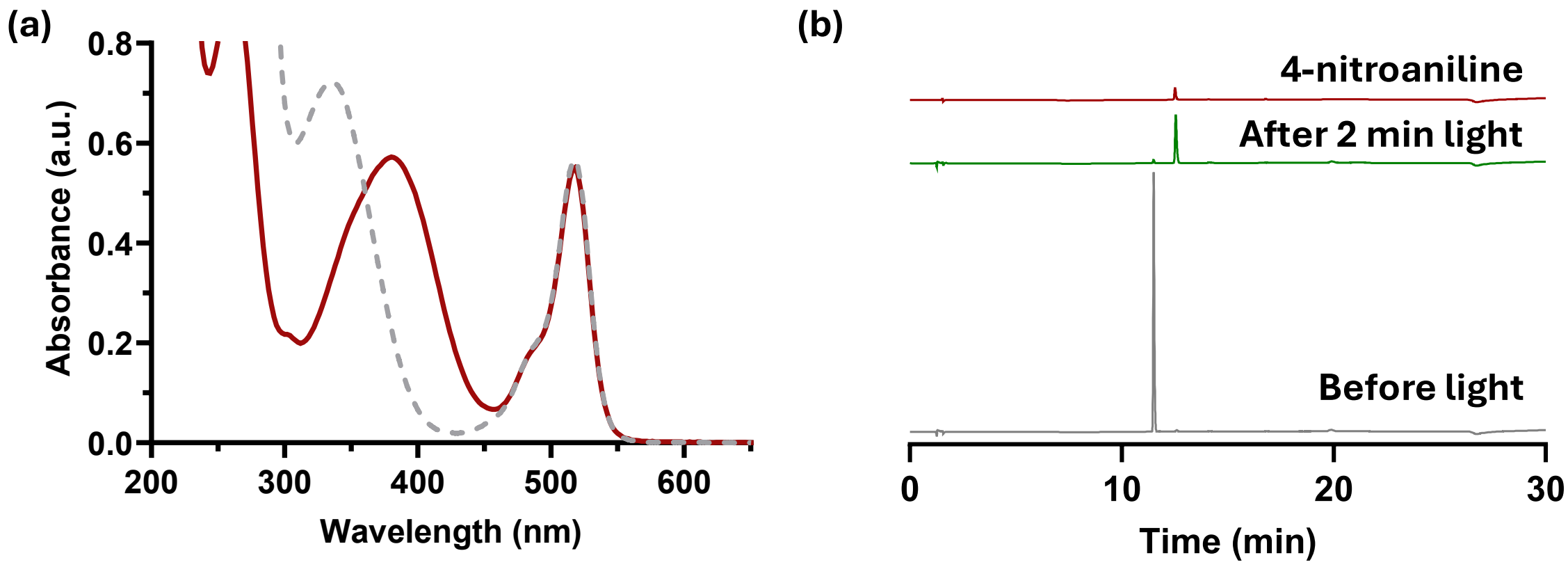

Figure S12. (a) Absorption spectra of Carbo-4NP (50 μM) containing NaAsc (400 μM) and Eosin Y (5 μM) in DI water before (grey dashed) and after 2 min green light irradiation (red). (b) HPLC traces of Carbo-4NP (300 μM) in water containing Eosin Y (30 μM) and NaAsc (2.4 mM) before and after 2 min green light irradiation (absorption at 320 nm).

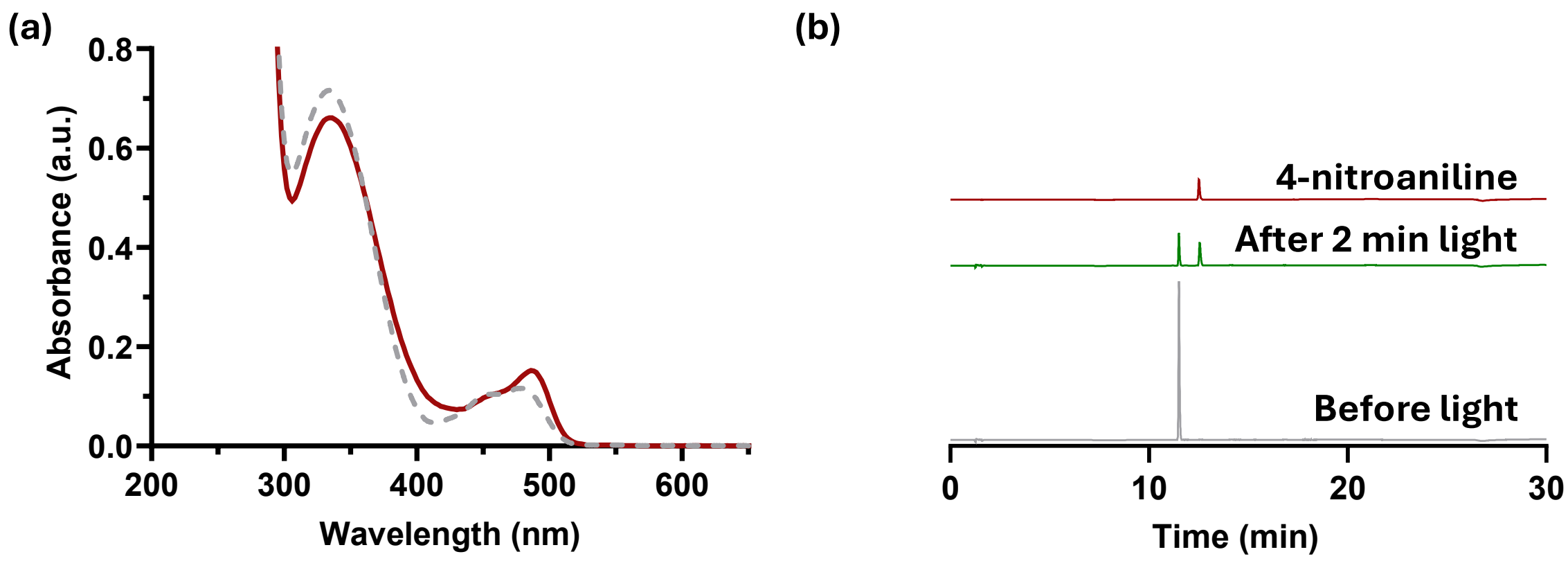

Figure S13. (a) Absorption spectra of Carbo-4NP (50 μM) containing NaAsc (400 μM) and Fluorescein (5 μM) in DI water before (grey dashed) and after 2 min green light irradiation (red). (b) HPLC traces of Carbo-4NP (300 μM) in water containing Fluorescein (30 μM) and NaAsc (2.4 mM) before and after 2 min green light irradiation (absorption at 320 nm).

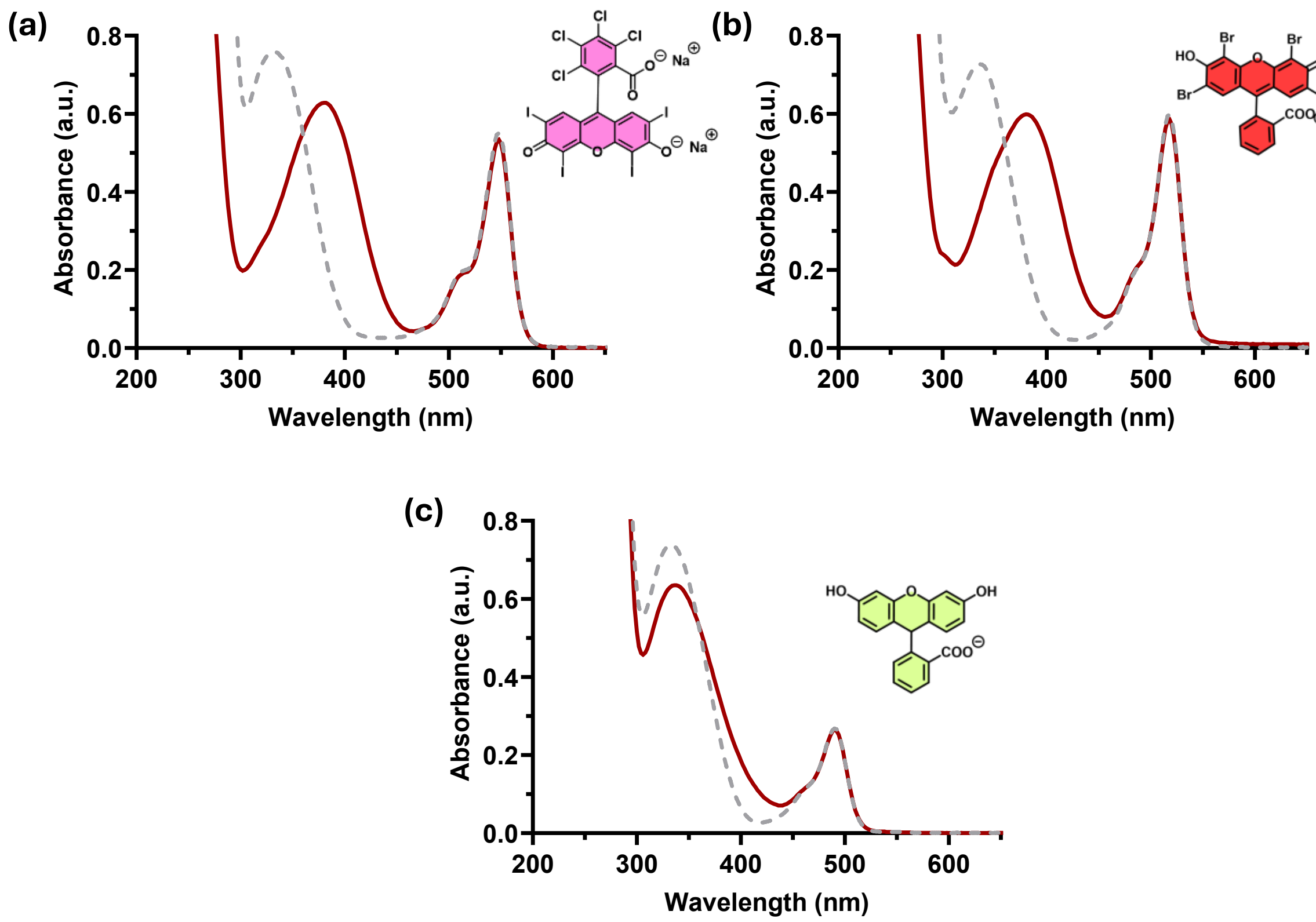

Figure S14. Absorption spectra of Carbo-4NP (50 μM) before (grey dashed) and after green light irradiation (red) in PBS pH 7.4 using (a) Rose Bengal, (b) Eosin Y and (c) Fluorescein as photocatalysts (5 μM) in presence of NaAsc (400 μM).

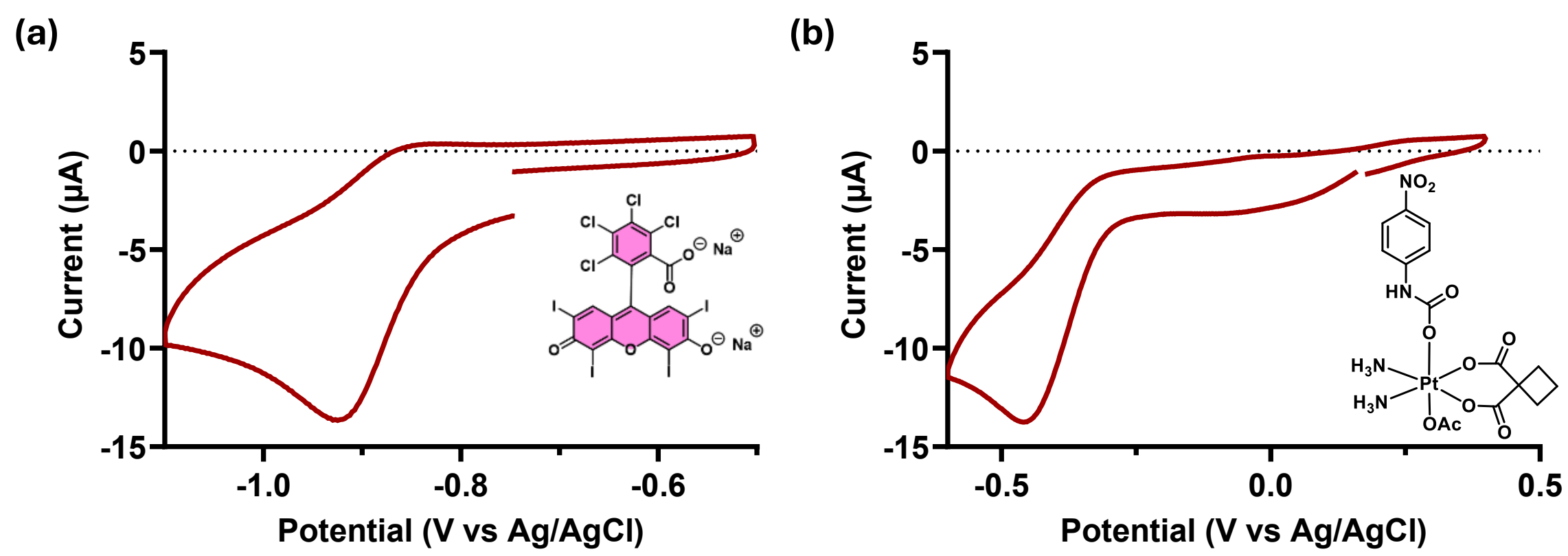

Figure S15. Cyclic Voltammogram of a 1 mM solution of (a) Rose Bengal and (b) 1 mM Carbo-4NP in PBS buffer (pH 7.40, with 10 % DMF) at a scan rate of 0.05 V/s.

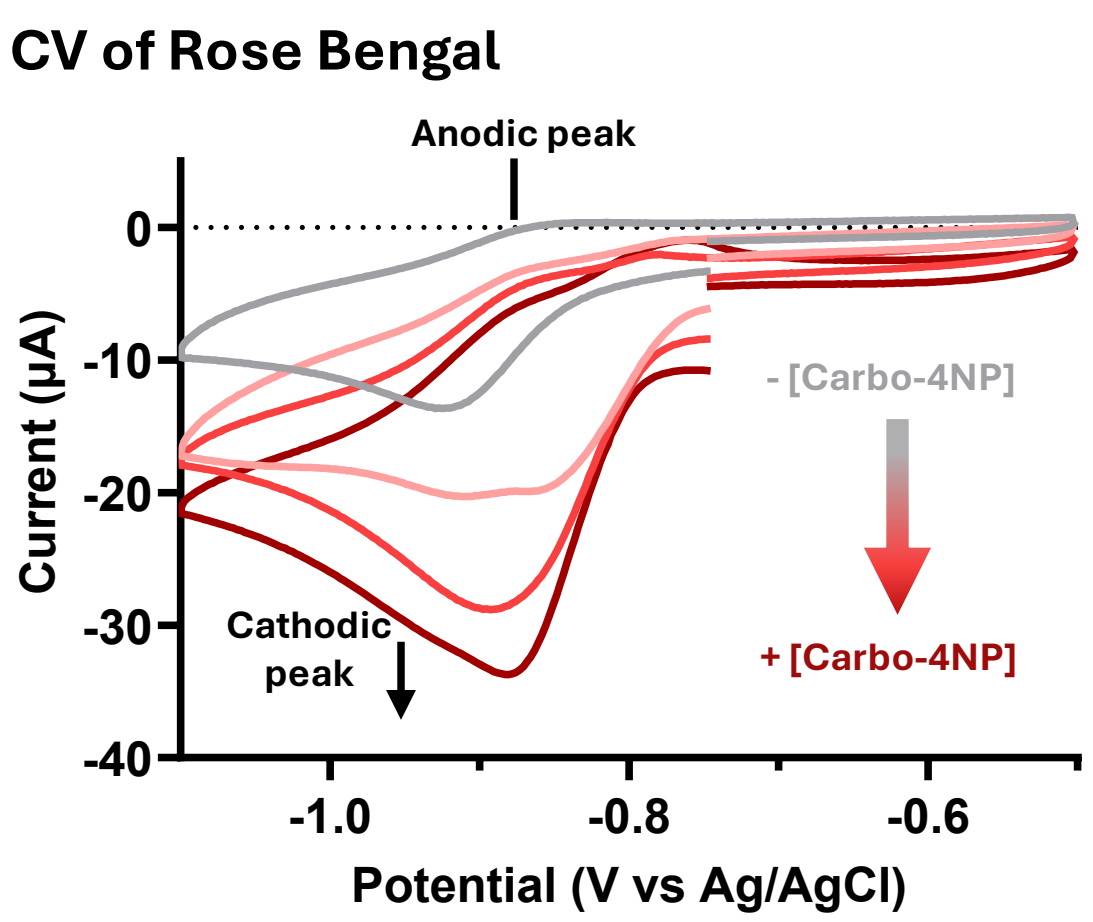

Figure S16. Cyclic Voltammogram of 1 mM Rose Bengal containing increasing concentrations of Carbo-4NP (0 mM (grey), 0.25 mM (light red), 0.5 mM (red), 1 mM (dark red)) in PBS buffer (pH 7.40, with 10 % DMF) at a scan rate of 0.05 V/s.

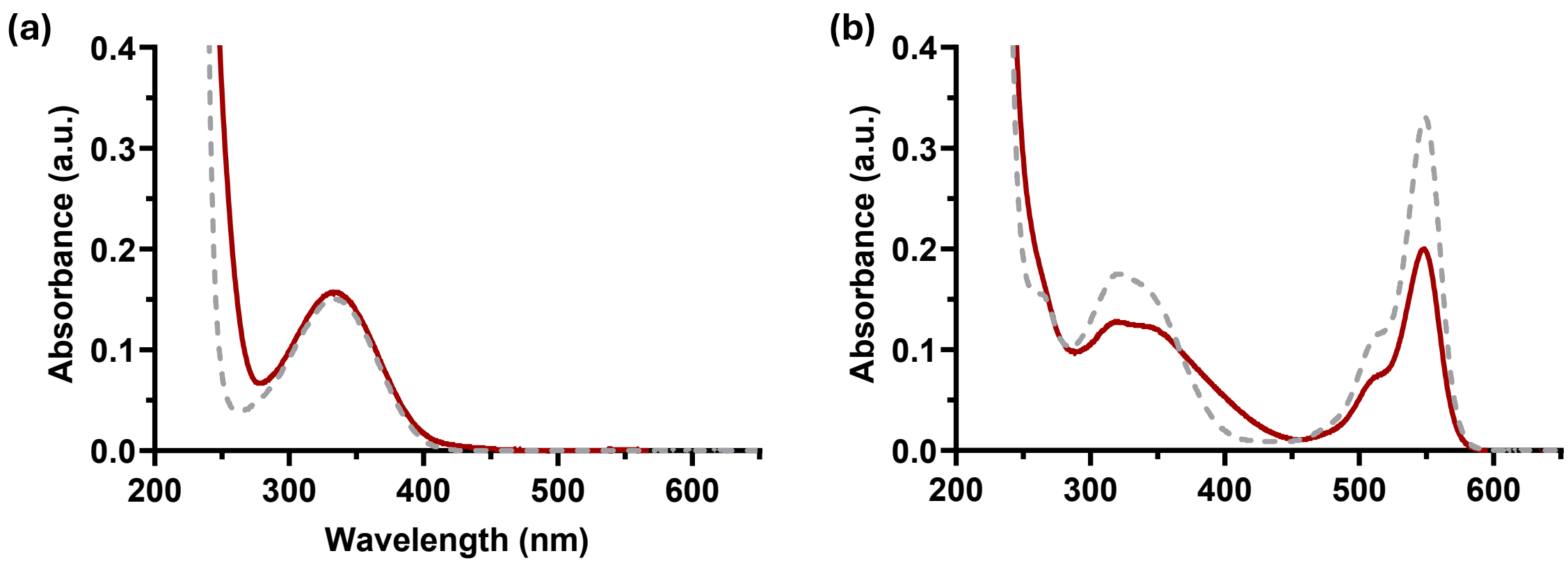

Figure S17. (a) Absorption spectra of Carbo-4NP (10 μM) before (grey dashed) and after electrochemical potential of -0.95 V was applied (red) in 0.1 M HEPES buffer pH 7.4. (b) Absorption spectra of Carbo-4NP (10 μM) containing 2.5 μM Rose Bengal before (grey dashed) and after electrochemical potential of -0.95 V was applied (red) in 0.1 M HEPES buffer pH 7.4.

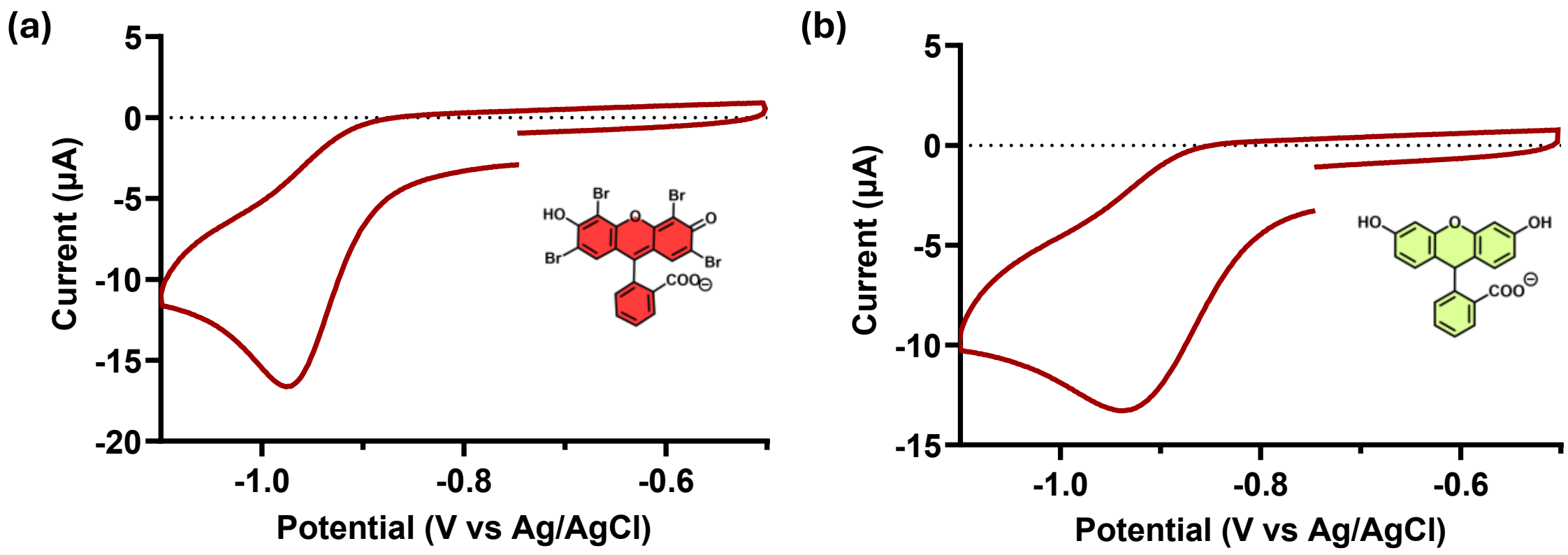

Figure S18. Cyclic Voltammogram of (a) 1 mM Eosin Y and (b) 1 mM Fluorescein in PBS buffer (pH 7.40, with 10 % DMF) at a scan rate of 0.05 V/s.

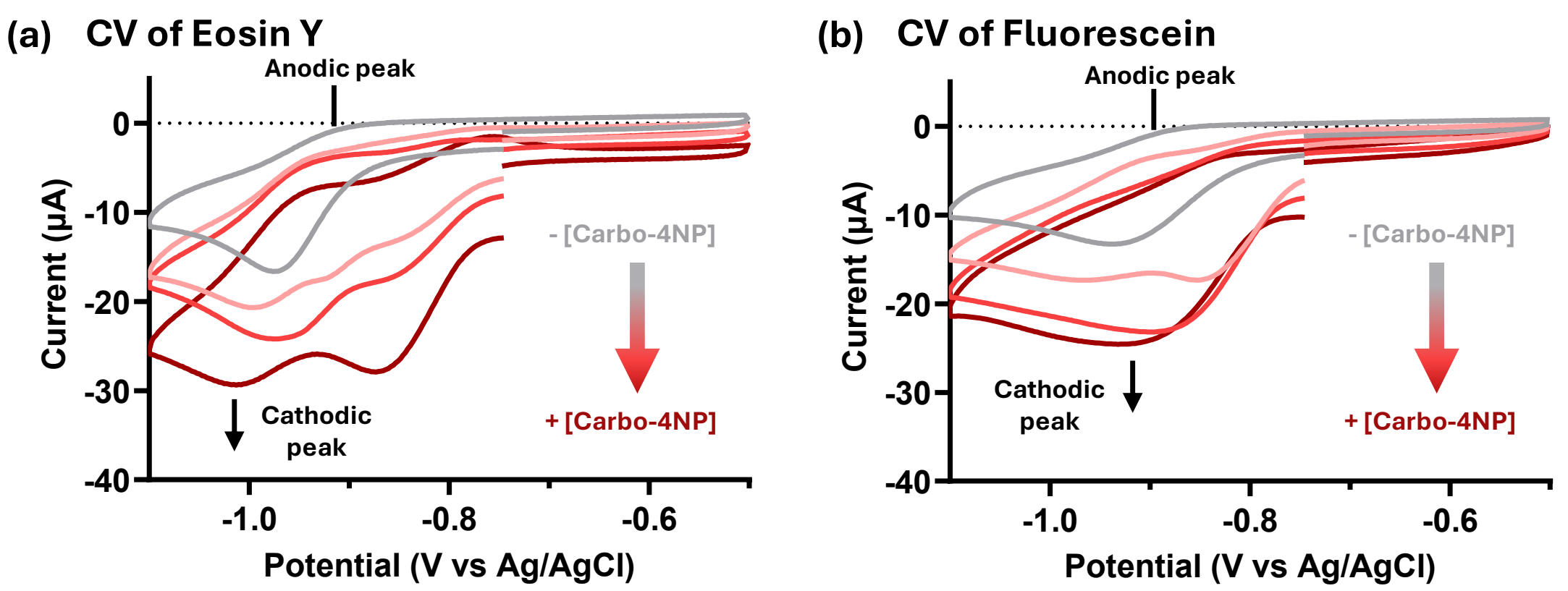

Figure S19. Cyclic Voltammogram of (a) 1 mM Eosin Y and (b) 1 mM Fluorescein, containing increasing concentrations of Carbo-4NP (0 mM (grey), 0.25 mM (light red), 0.5 mM (red), 1 mM (dark red)) in PBS buffer (pH 7.40, with 10 % DMF) at a scan rate of 0.05 V/s.

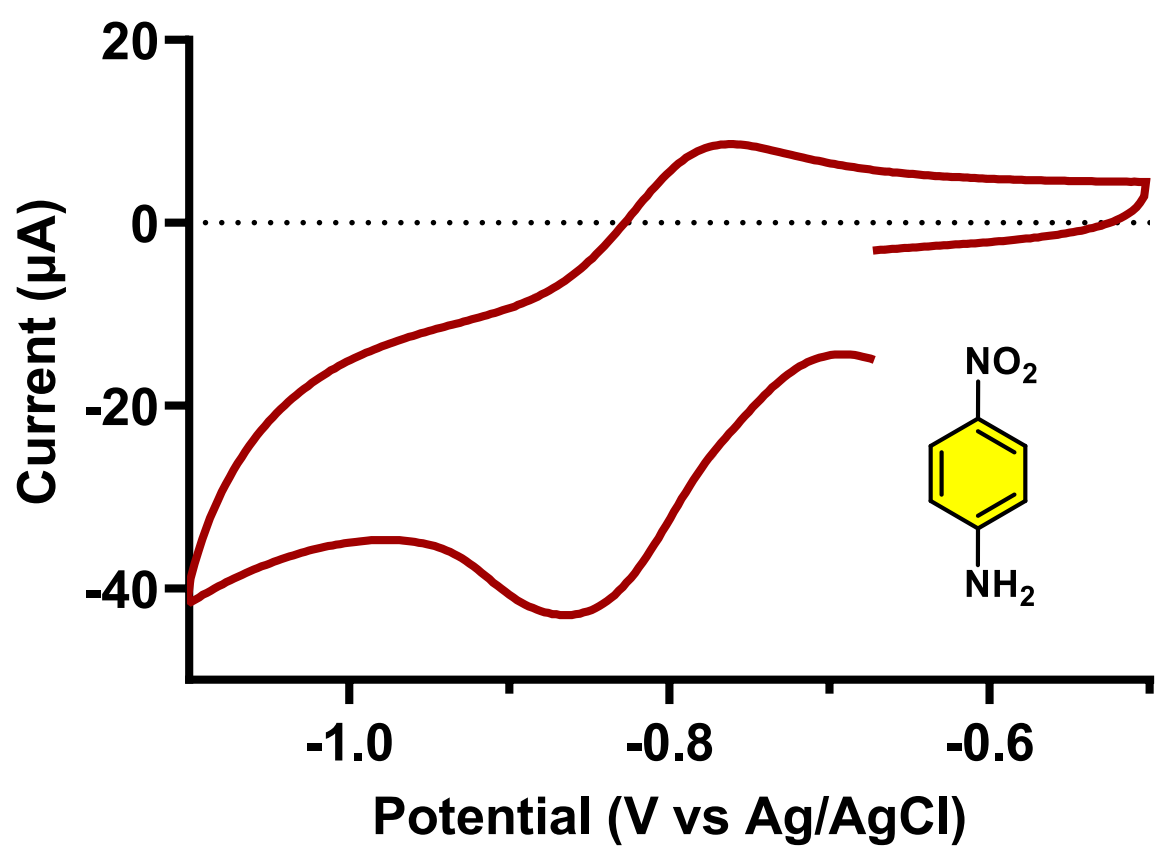

Figure S20. Cyclic Voltammogram of 1 mM 4-nitroaniline in PBS buffer (pH 7.40, with 10 % DMF) at a scan rate of 0.05 V/s.

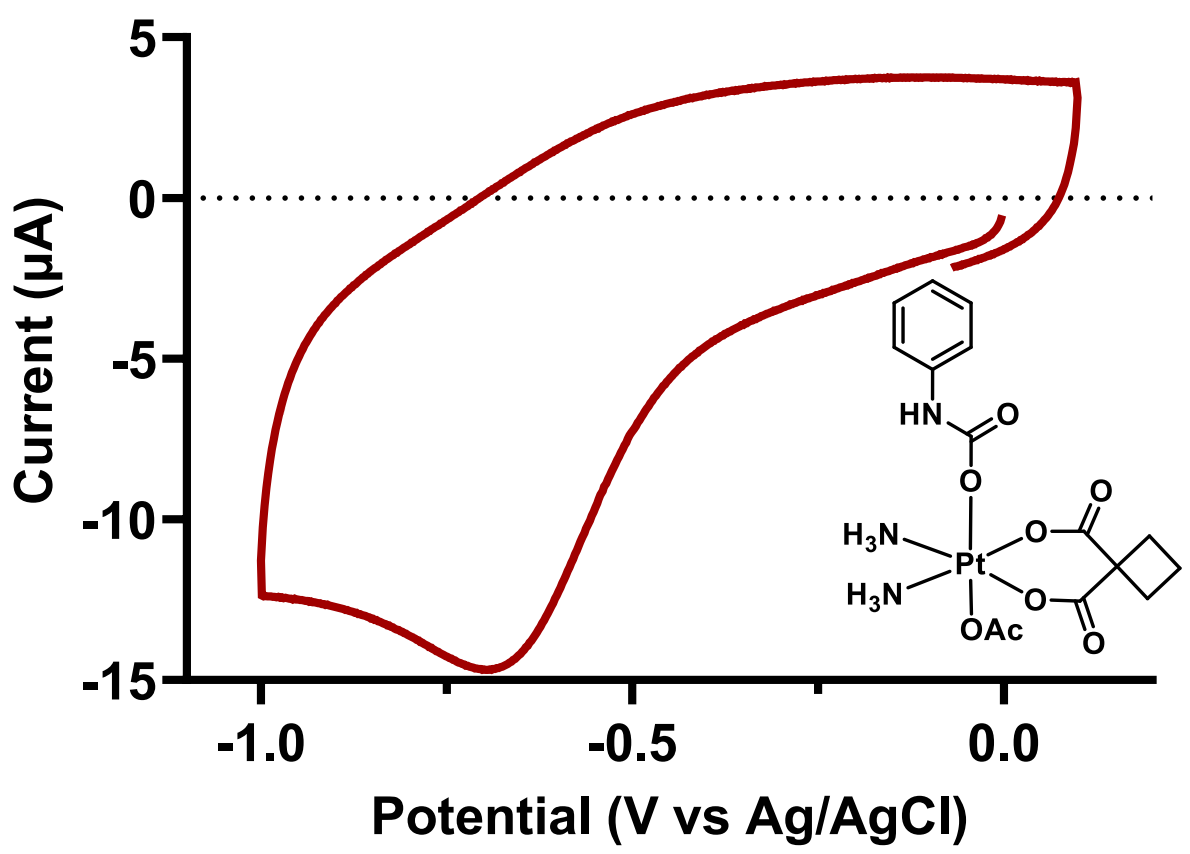

Figure S21. Cyclic Voltammogram of 1 mM CarboPhen in PBS buffer (pH 7.40, with 10 % DMF) at a scan rate of 0.05 V/s.

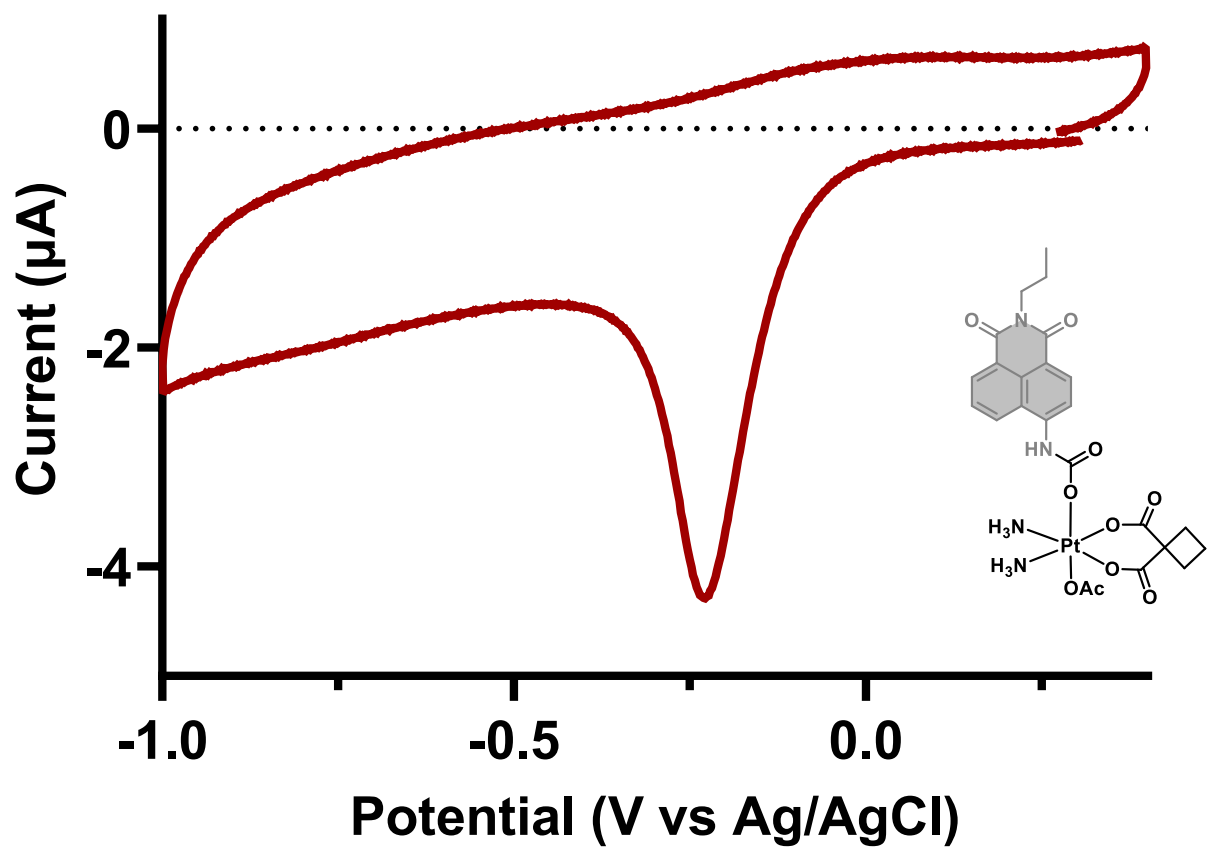

Figure S22. Cyclic Voltammogram of 1 mM CarboNap in PBS buffer (pH 7.40, with 10 % DMF) at a scan rate of 0.05 V/s.

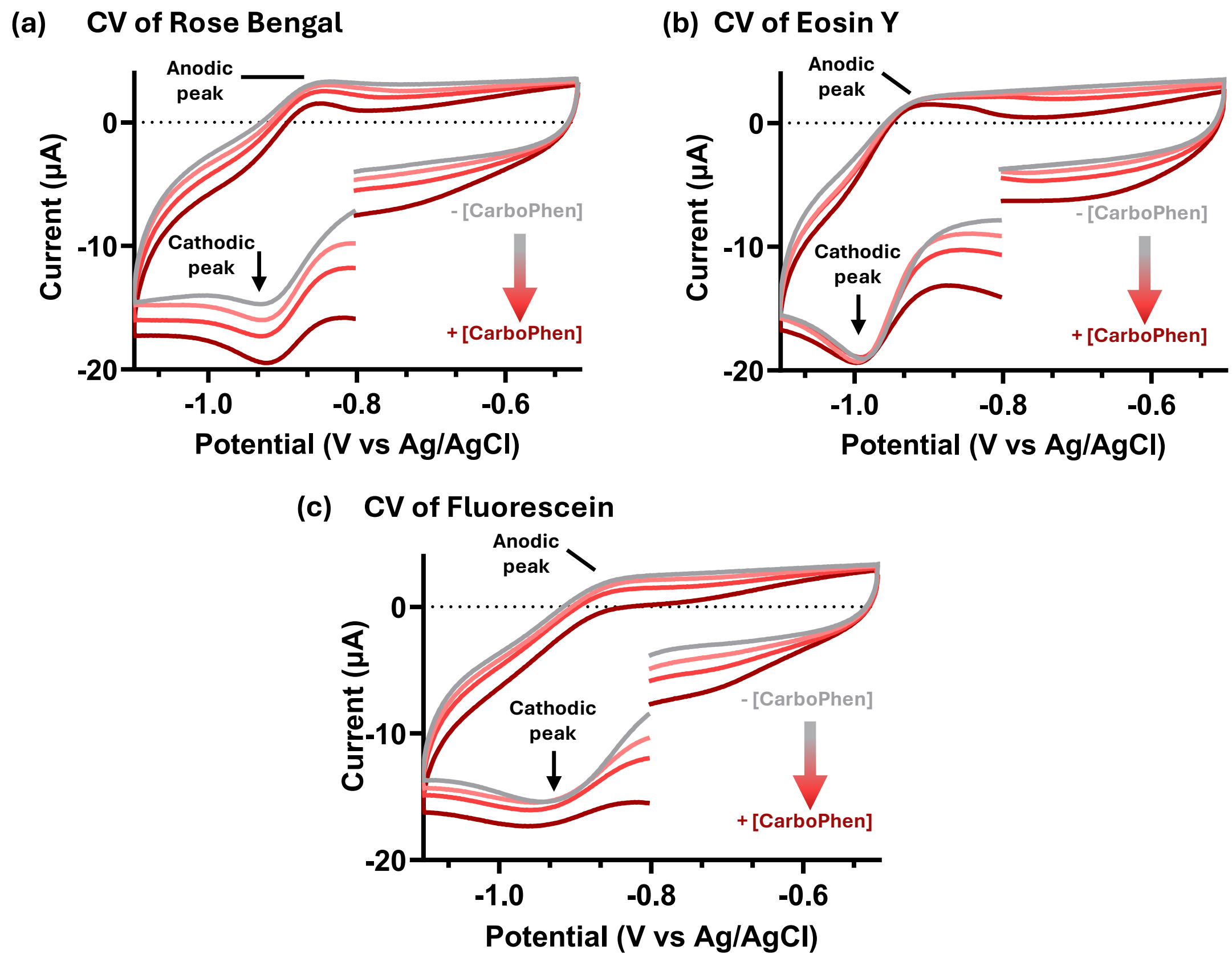

Figure S23. Cyclic Voltammogram of (a) 1 mM Rose Bengal, (b) 1 mM Eosin Y and (c) 1 mM Fluorescein, containing increasing concentrations of CarboPhen (0 mM (grey), 0.25 mM (light red), 0.5 mM (red), 1 mM (dark red)) in PBS buffer (pH 7.40, with 10 % DMF) at a scan rate of 0.05 V/s.

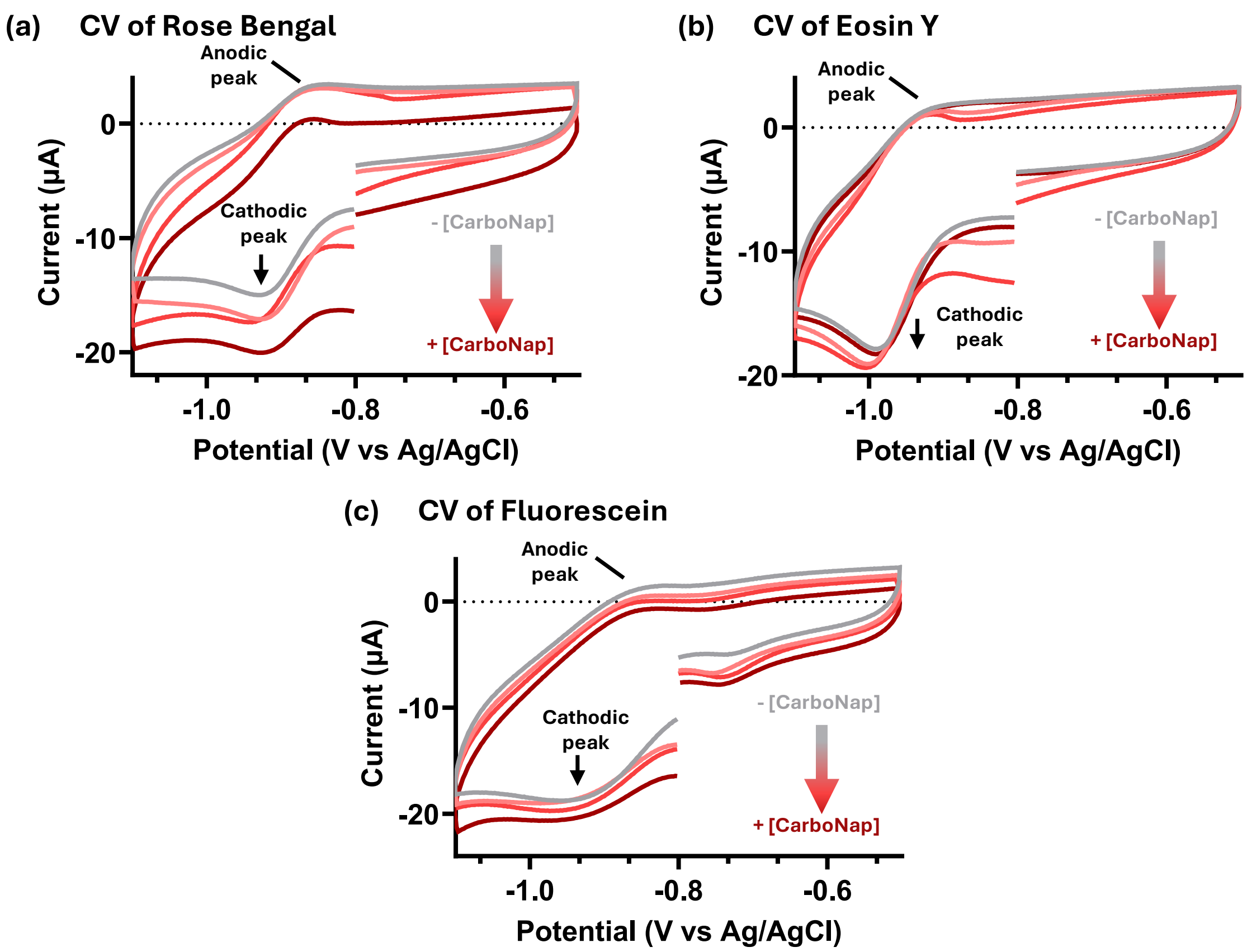

Figure S24. Cyclic Voltammogram of (a) 1 mM Rose Bengal, (b) 1 mM Eosin Y and (c) 1 mM Fluorescein, containing increasing concentrations of CarboNap (0 mM (grey), 0.25 mM (light red), 0.5 mM (red), 1 mM (dark red)) in PBS buffer (pH 7.40, with 10 % DMF) at a scan rate of 0.05 V/s.

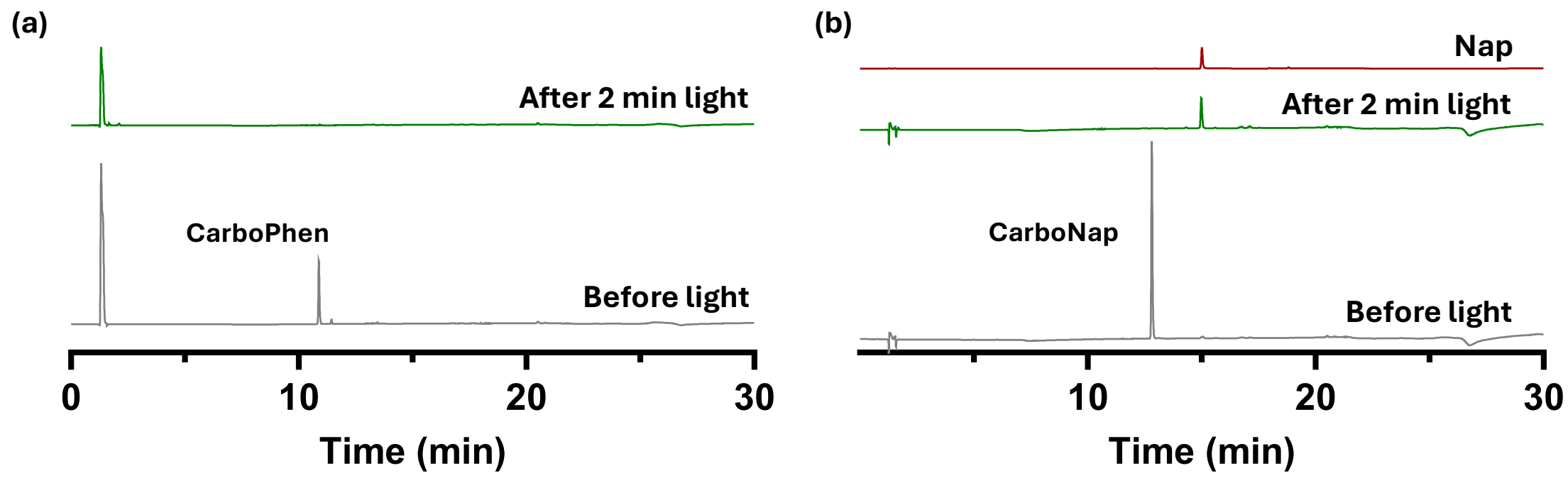

Figure S25. (a) HPLC traces of CarboPhen (300 µM) in water containing Rose Bengal (30 µM) and NaAsc (2.4 mM) before (grey) and after 2 min green light irradiation (green) (absorption at 272 nm) showing that the CarboPhen peak (R_t= 10.9 min) disappeared after light irradiation. Note - no aniline product was observed on HPLC spectrum. (b) HPLC traces of CarboNap (300 µM) in water containing Rose Bengal (30 µM) and NaAsc (2.4 mM) before (grey) and after 2 min green light irradiation (green) (absorption at 340 nm).

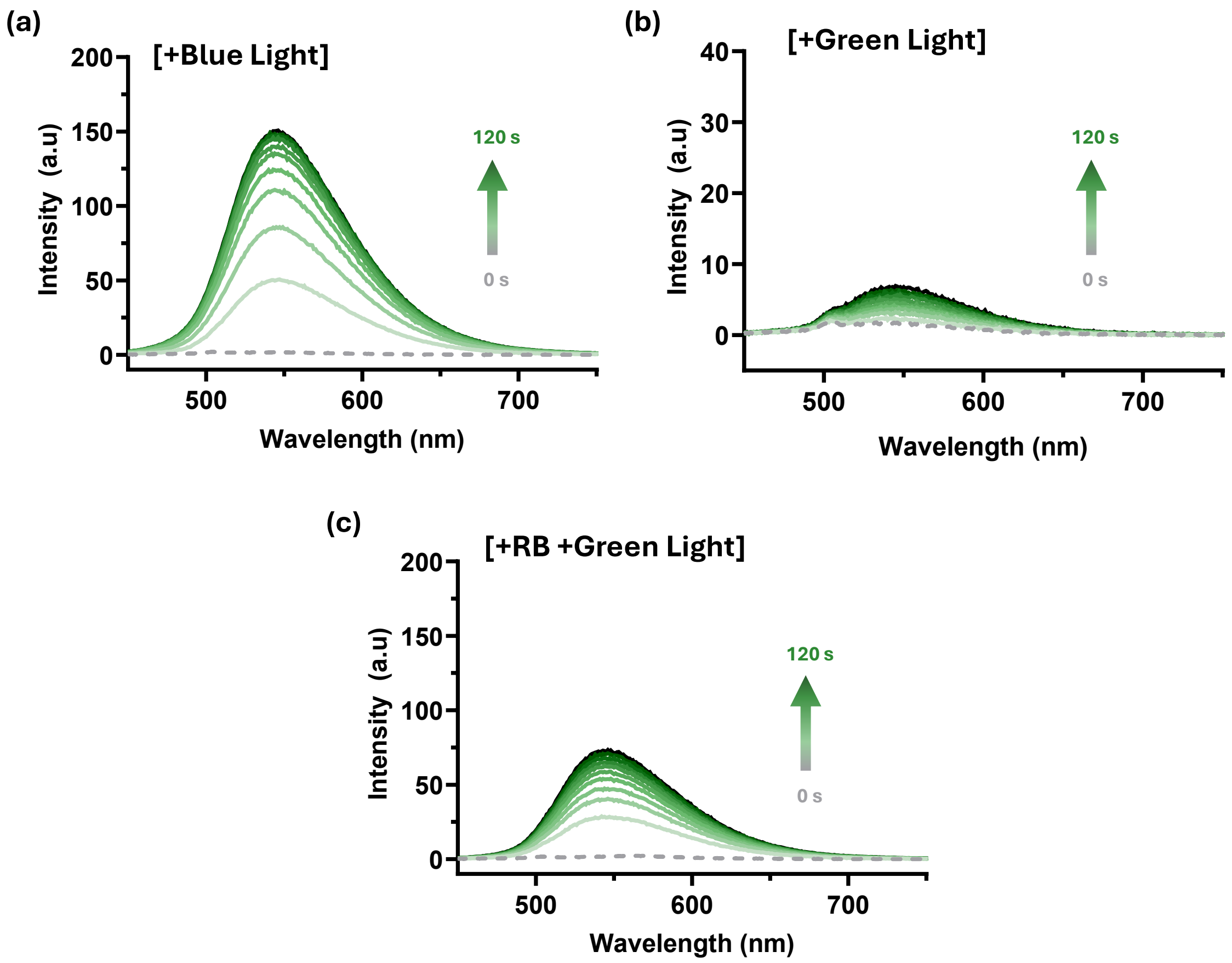

Figure S26. Fluorescence emission spectra of CarboNap (5 µM) after being irradiated with blue (a), green light (b) and green light in the presence of RB (0.5 µM) (c) from 0 (grey) to 120 sec (dark green). Excitation: 430, emission: 450-800 nm, Exc. slit: 5nm, Em. Slit: 5 nm.

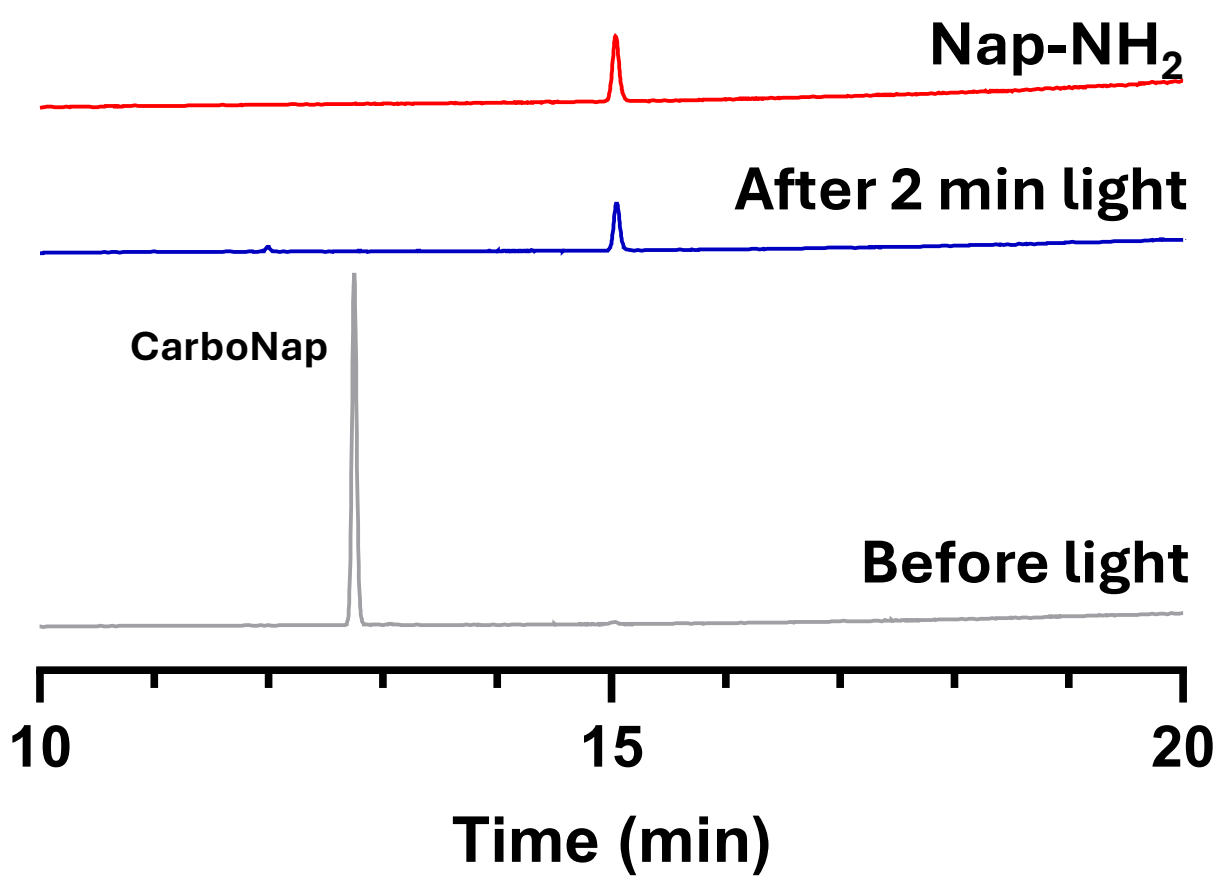

Figure S27. (a) HPLC traces of CarboNap (50 µM) in water containing Rose Bengal (5 µM) and NaAsc (0.4 mM) in 30:70 ACN:H_2O before and after 2 min green light irradiation (absorption at 320 nm).

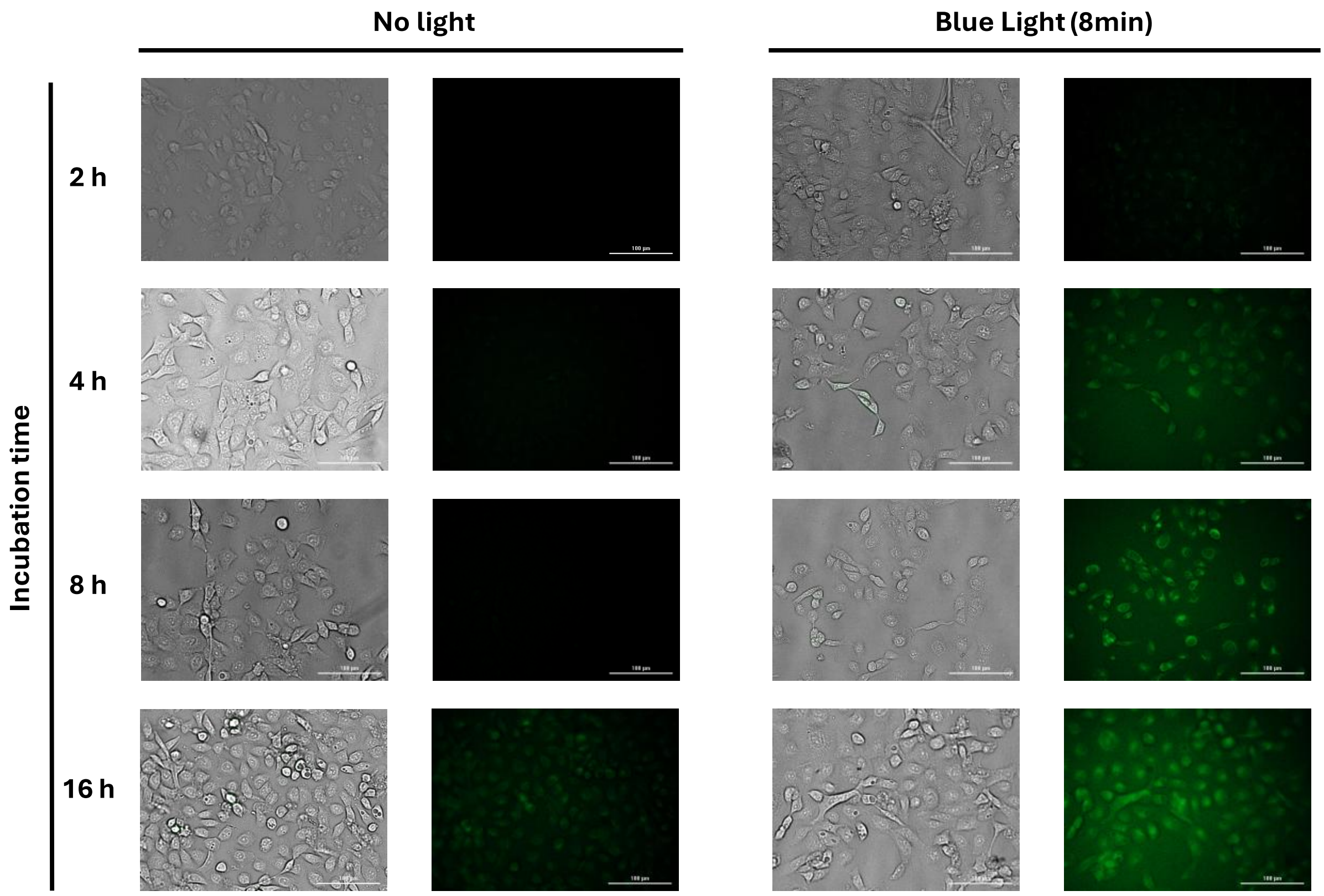

Figure S28. Fluorescent images of HN5 cells treated with CarboNap (10 µM) were incubated for 2 h, 4 h, 8 h and 16 h at 37 °C and then exposed to blue light for 8 min before analysis via cytation 5 microscopy, with representative pictures shown (scale bar = 100 µm).

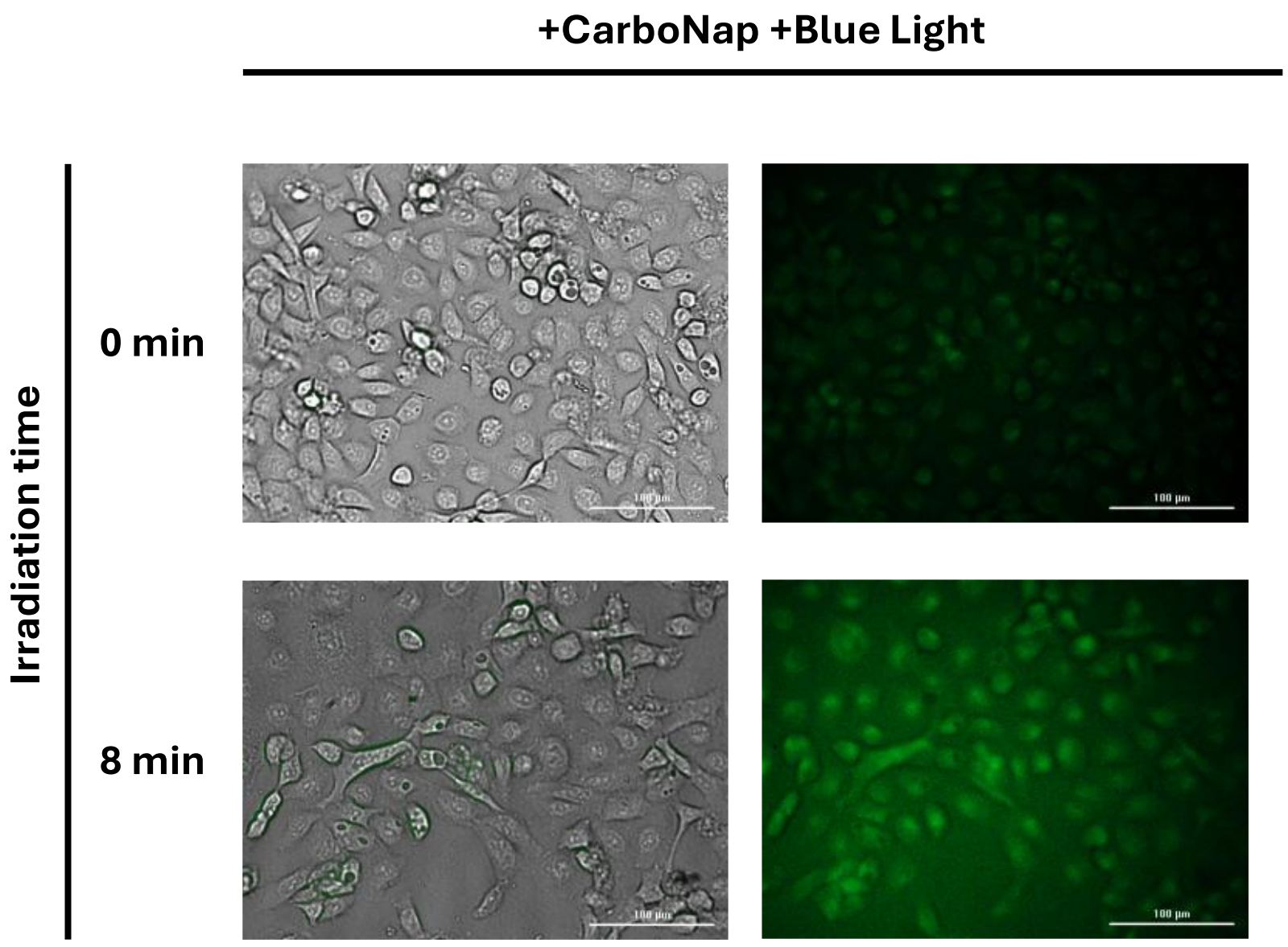

Figure S29. Fluorescent images of HN5 cells treated with CarboNap (10 µM) were incubated overnight at 37 °C and then exposed to blue light for 0 and 8 min before analysis via Cytation 5, with representative pictures shown (scale bar = 100 µm).

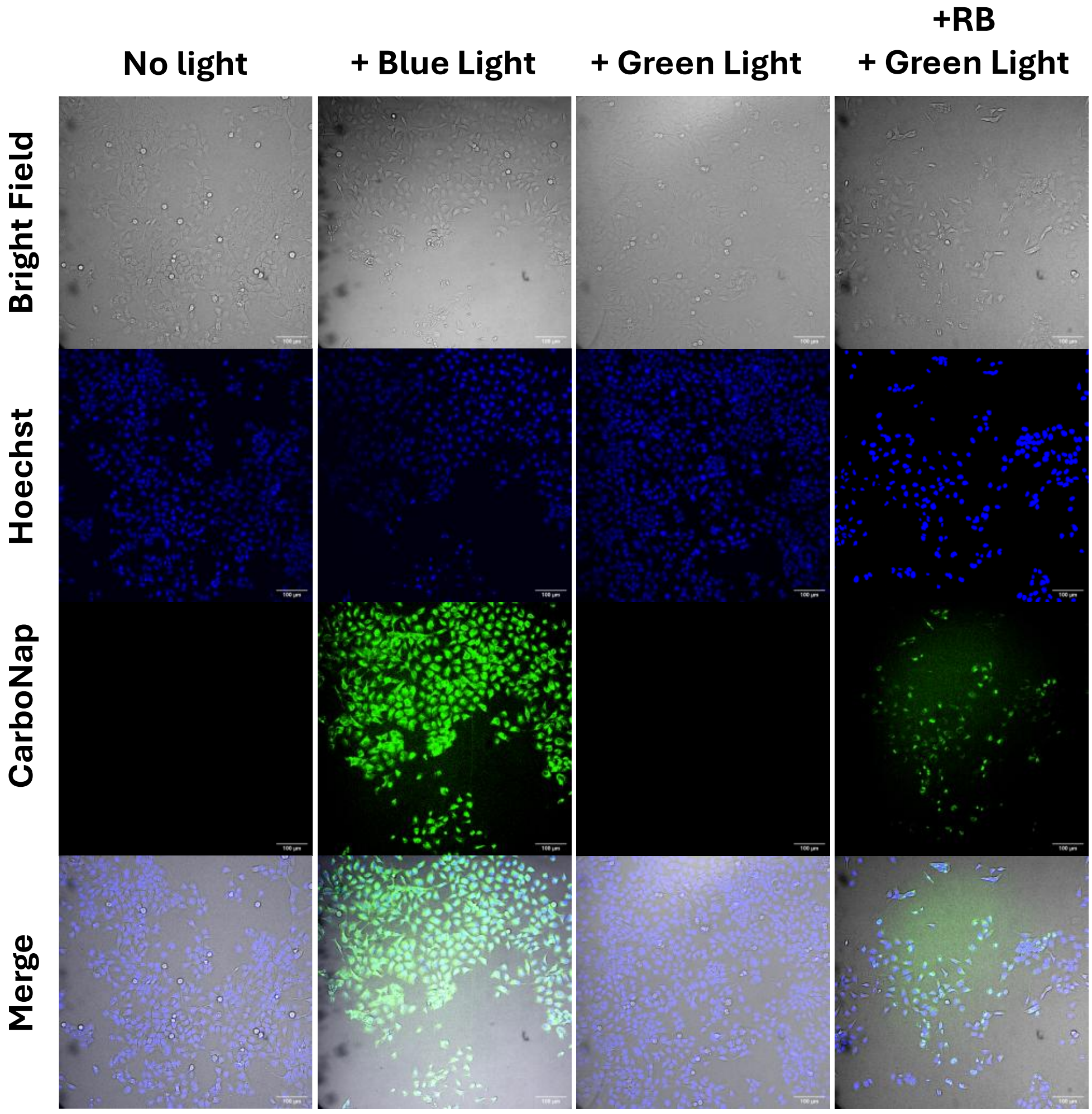

Figure S30. Confocal images of HN5 cells treated with CarboNap (10 μM) were incubated overnight at 37 °C in the presence and absence of Rose Bengal (12.5 μM) and then exposed to blue light or green light of 8 min. Pictures were taken with SoRa confocal, with representative pictures shown (scale bar = 100 μm).

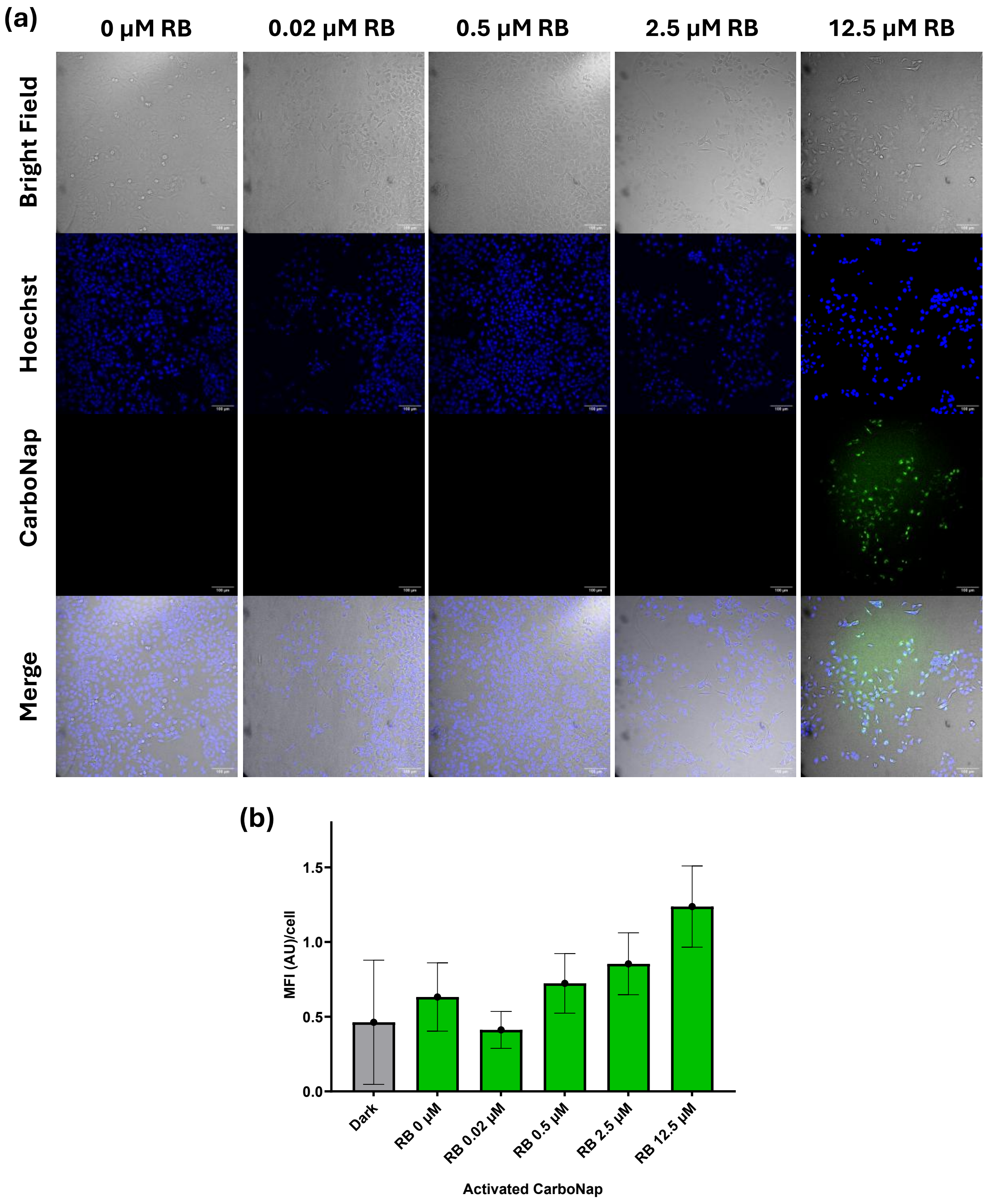

Figure S31. (a) Confocal images of HN5 cells treated with CarboNap (10 μM) were incubated overnight at 37 °C with different concentrations of Rose Bengal (0.02, 0.5, 2.5 and 12.5 μM) and then exposed to green light for 8 min. Pictures were taken with SoRa confocal, with representative pictures shown (scale bar = 100 μm). (b) Fluorescence intensity of HN5 cell containing CarboNap (10 μM) with different concentrations of RB before (grey) and after green light irradiation (green). Each picture was quantified using Fiji. Data shown in b is presented as mean of 10 individual cells + SD.

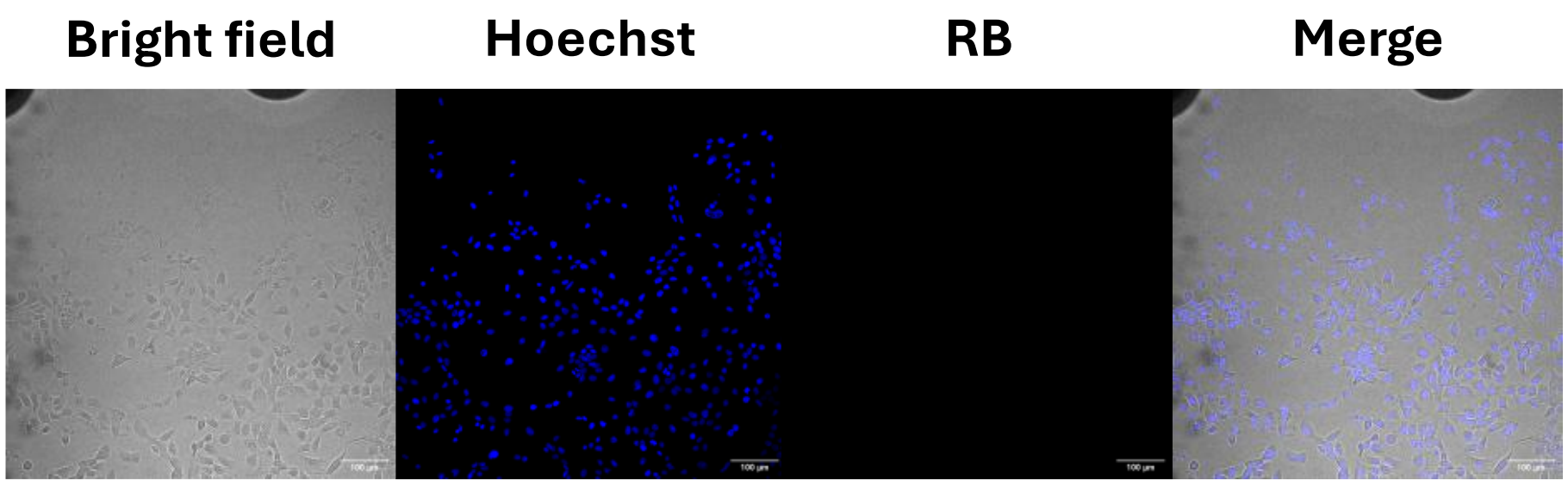

Figure S32. Confocal images of HN5 cells treated with Rose Bengal (12.5 µM) were incubated overnight at 37 °C and then exposed to green light for 8 min. Pictures were taken with SoRa confocal, with representative pictures shown (scale bar = 100 µm).

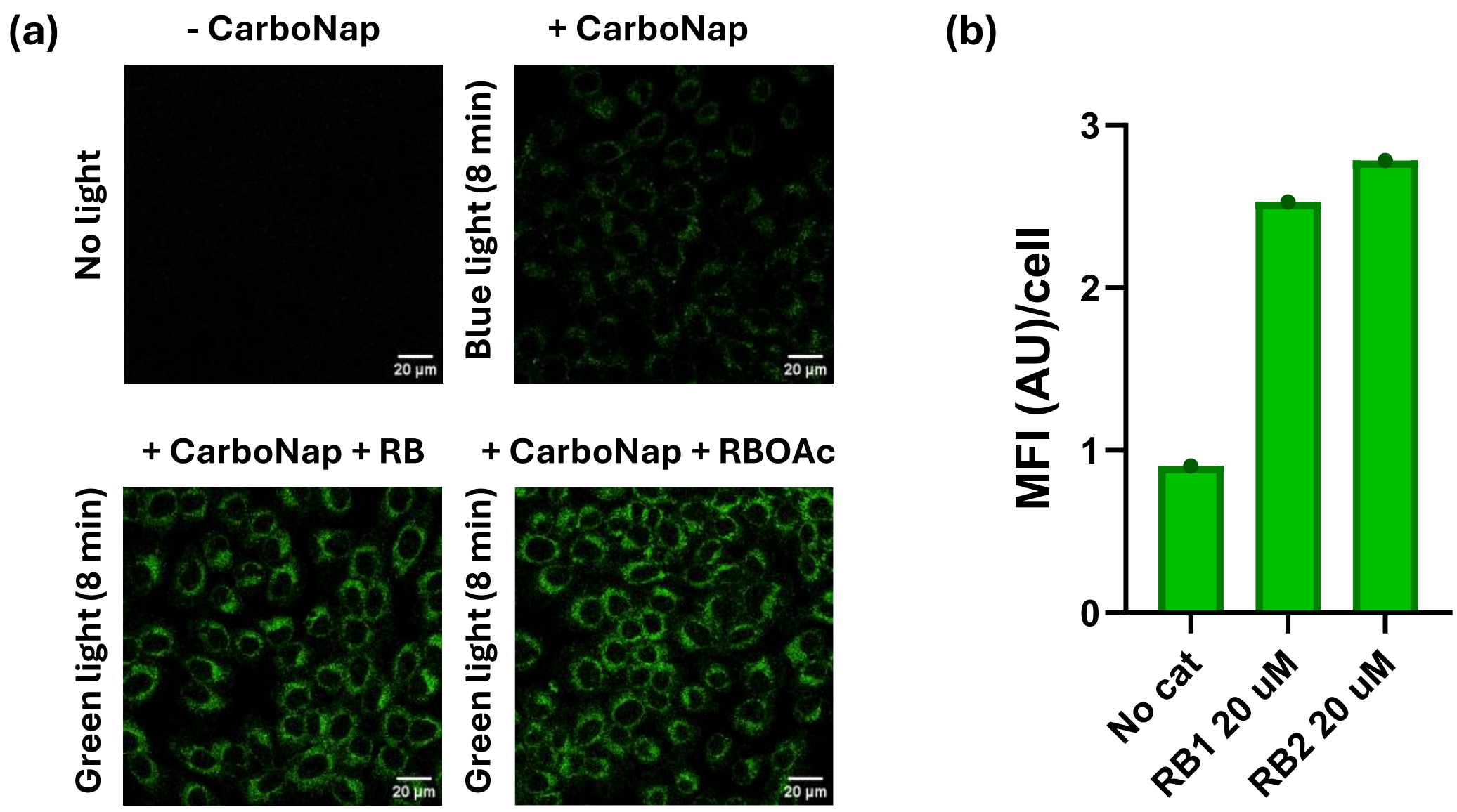

Figure S33. (a) Confocal images of HN5 cells treated with CarboNap (10 µM) and Rose Bengal (RB1) (20 µM) or Rose bengal diacetate (RB2) (20 µM) were incubated overnight at 37 °C and then exposed to green light for 8 min before analysis via Nikon AXR point scanning confocal at 60x with an oil immserion objective. Representative pictures shown (scale bar = 20 µm). (b) Fluorescence intensity quantified for each picture using Fiji. Data shown in b is presented as mean of 10 individual cells.

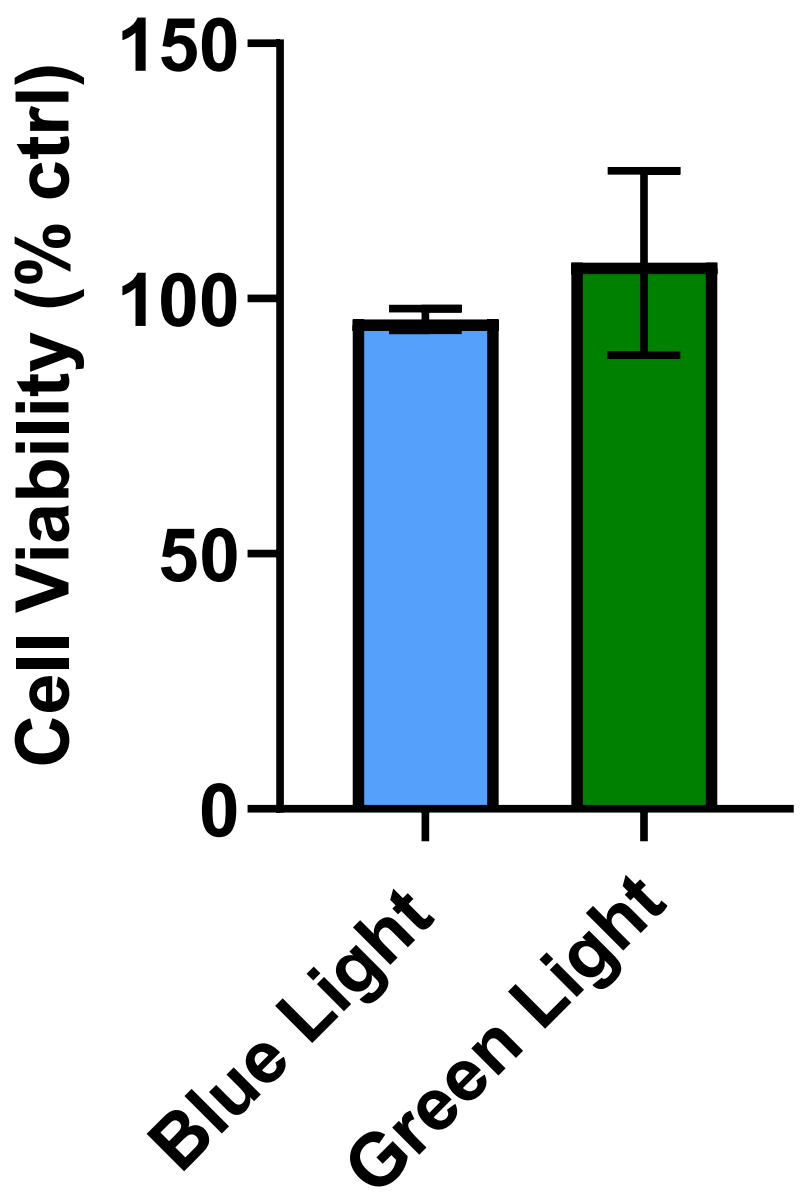

Figure S34. Cell cytotoxicity of HN5 cells incubated overnight in media containing 0.1 % DMSO irradiated for 8 min using blue and green light, and after incubating for an extra 24 h. Data are shown as percentage viability relative to the dark control.

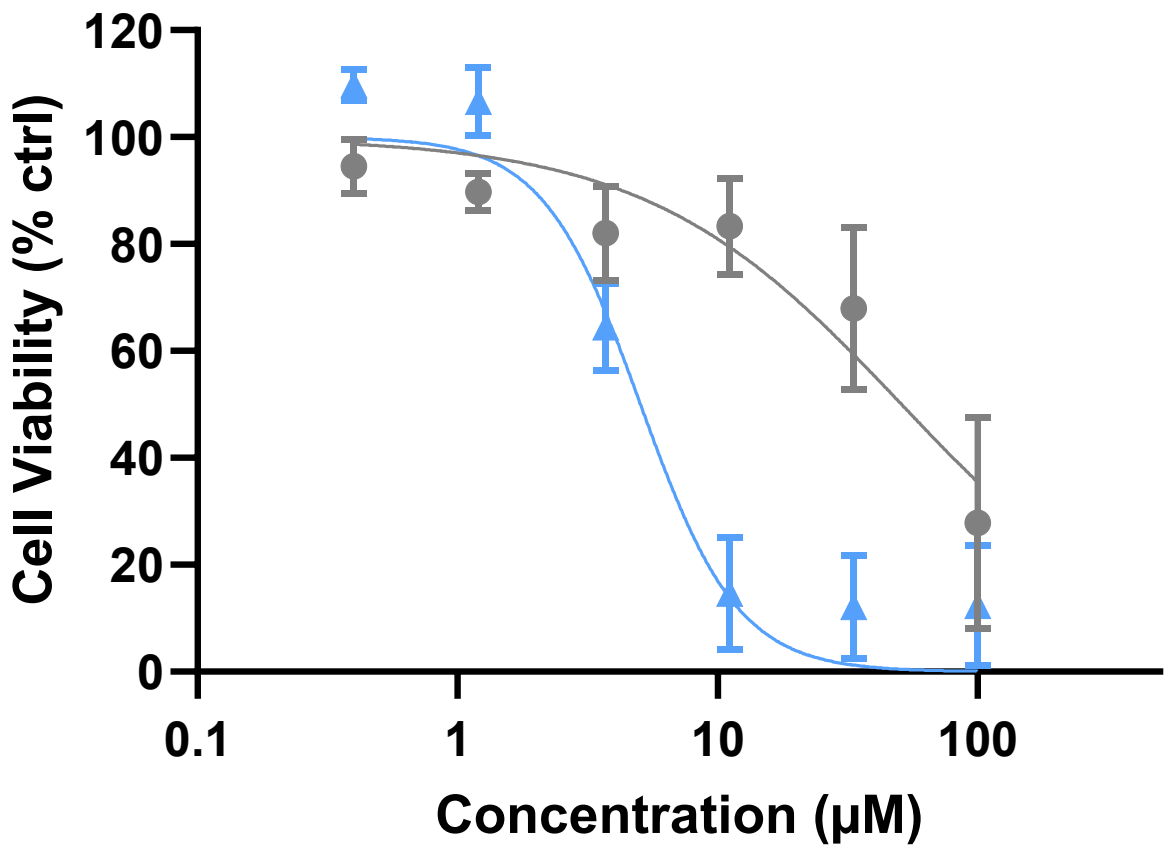

Figure S35. Cell cytotoxicity of HN5 cells incubated overnight with different concentrations of CarboNap, irradiated for 0 (grey) and 8 min (blue) with blue light and after incubating for an extra 24 h.

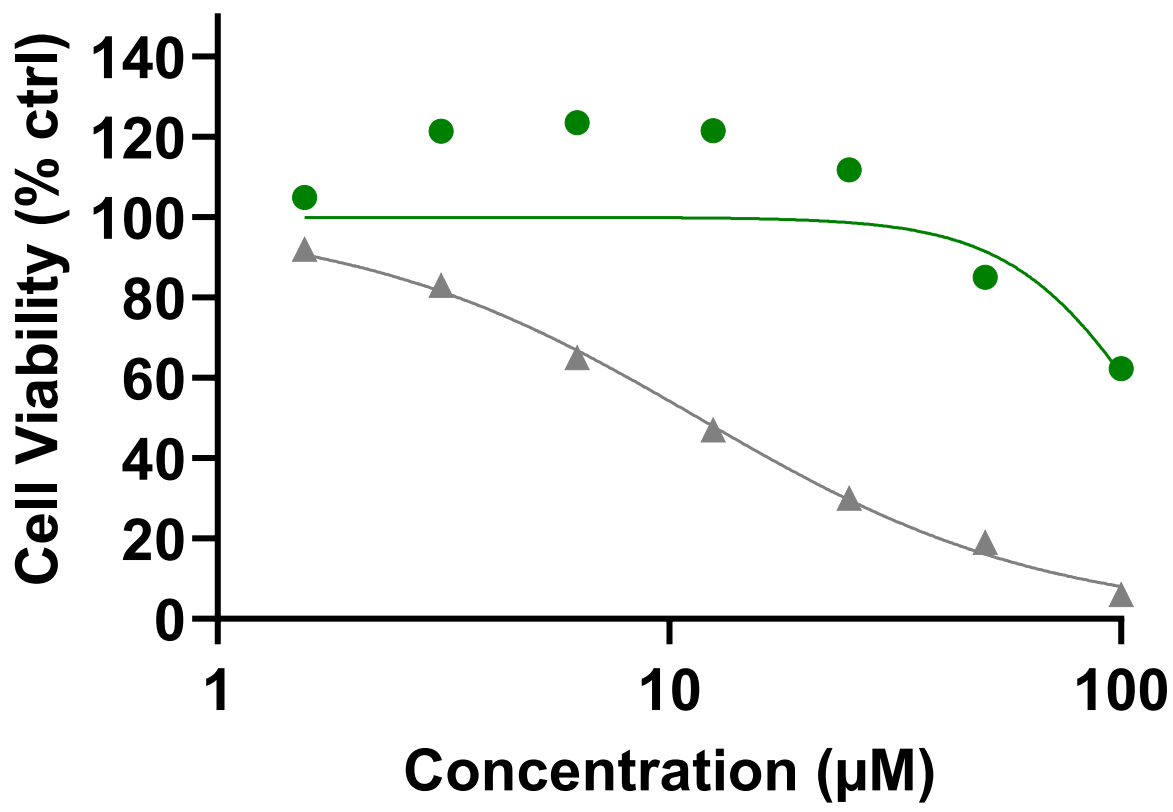

Figure S36. Cell cytotoxicity of HN5 cells incubated overnight with different concentrations of Rose Bengal, irradiated for 0 (grey) and 8 min (green) with green light (520 nm, 17.8 mW), and after incubating for an extra 24 h.

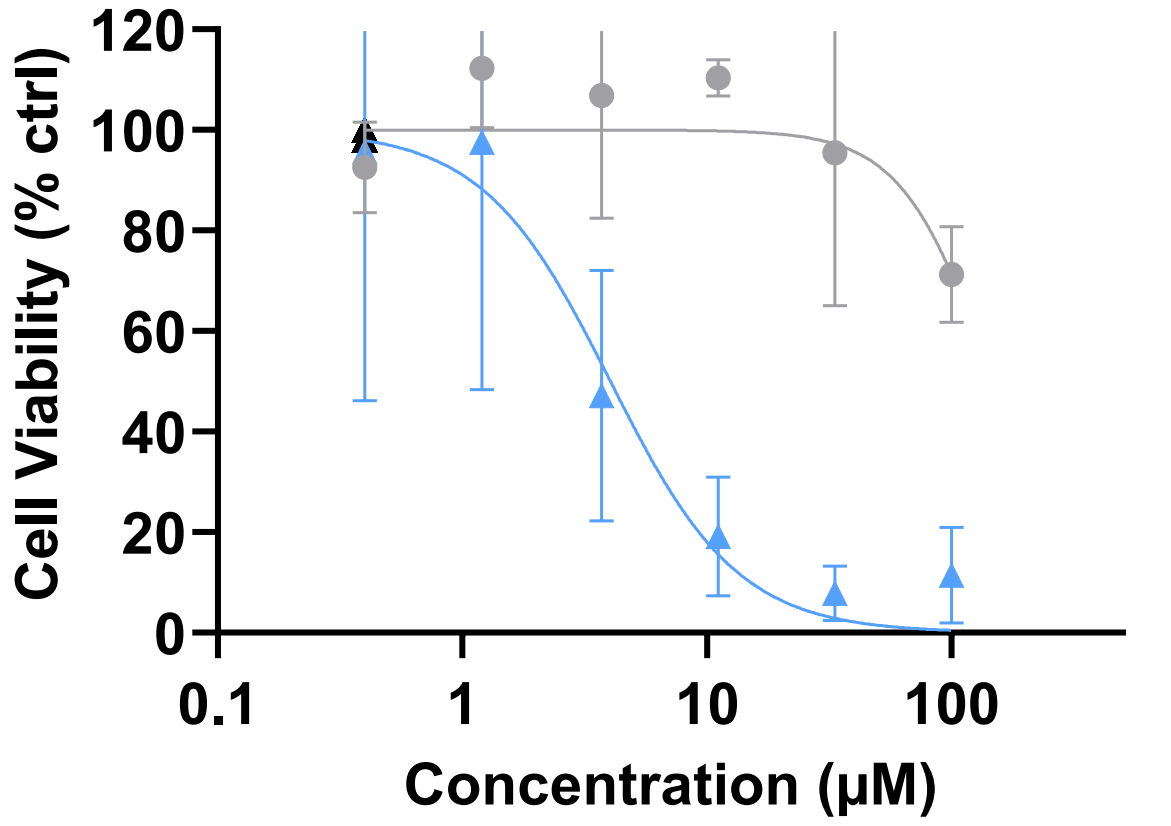

Figure S37. Cell cytotoxicity of MDA-231 cells co-incubated overnight with different concentrations of CarboNap, irradiated for 0 (grey) and 8 min (green) with blue light and after incubating for an extra 24 h.

4. NMR spectra

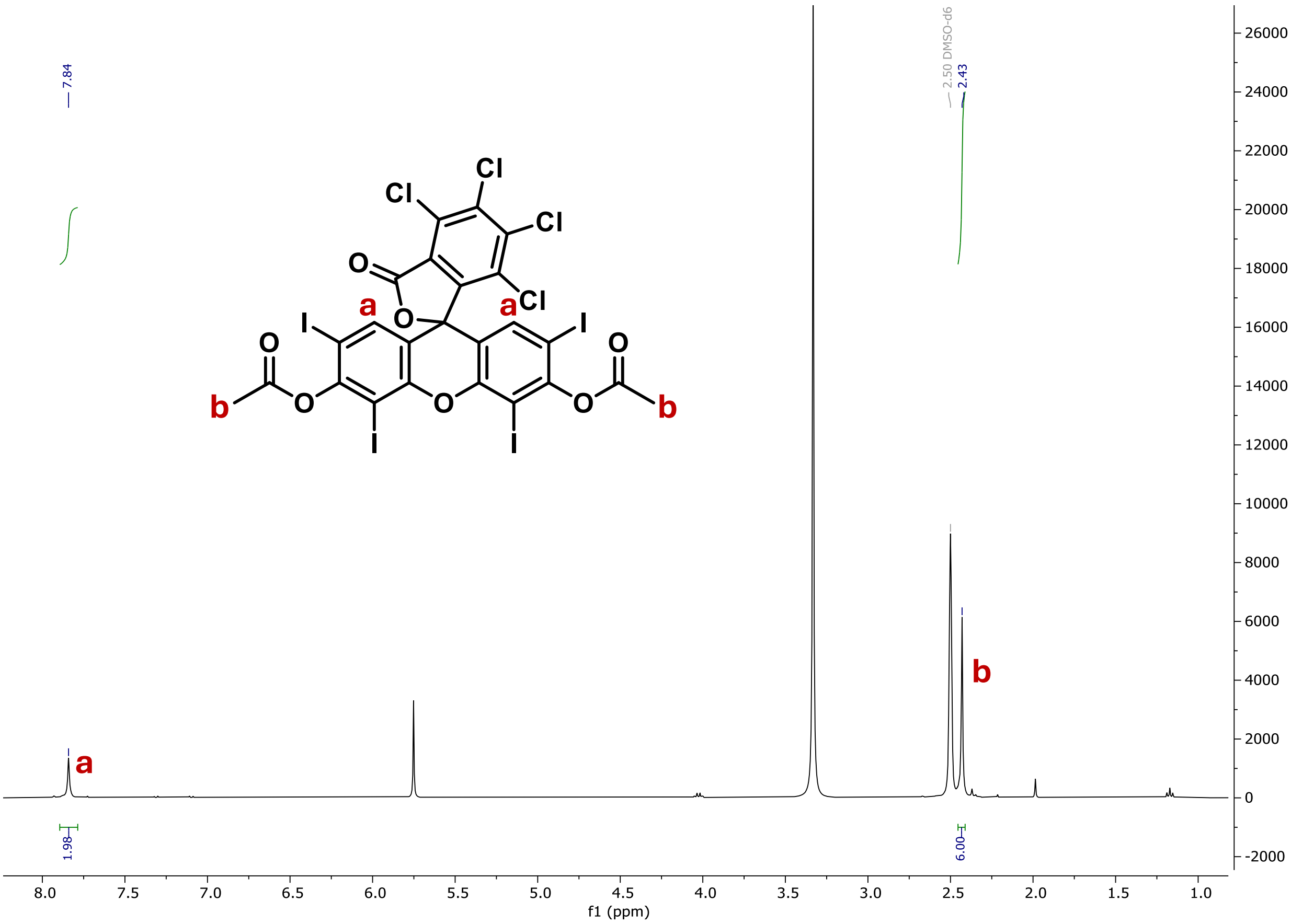

Figure S38 ^{1}H NMR spectrum (400 MHz, 25 °C, DMSO-d_6) of rose bengal diacetate.

5. References

1. J. W. Marsh, L. Hacker, S. Huang, M. H. C. Boulet, J. R. G. White, L. A. W. Martin, M. A. Yeomans, H.-H. Han, I. Diez-Perez, R. A. Musgrave, E. M. Hammond and A. C. Sedgwick, *Dalton Trans.*, 2024, **53**, 14811-14816.
2. G. Bottiroli, A. C. Croce, P. Balzarini, D. Locatelli, P. Baglioni, P. Lo Nostro, M. Monici and R. Pratesi, *Photochem. Photobiol.*, 1997, **66**, 374-383.
